# *In vivo* human neurite exchange time imaging at 500 mT/m diffusion gradients

**DOI:** 10.1162/imag_a_00544

**Published:** 2025-04-22

**Authors:** Kwok-Shing Chan, Yixin Ma, Hansol Lee, José P. Marques, Jonas L. Olesen, Santiago Coelho, Dmitry S. Novikov, Sune N. Jespersen, Susie Y. Huang, Hong-Hsi Lee

**Affiliations:** Athinoula A. Martinos Center for Biomedical Imaging, Charlestown, MA, United States; Department of Radiology, Massachusetts General Hospital, Harvard Medical School, Boston, MA, United States; Donders Institute for Brain, Cognition and Behaviour, Radboud University, Nijmegen, The Netherlands; Center of Functionally Integrative Neuroscience (CFIN) and MINDLab, Department of Clinical Medicine, Aarhus University, Aarhus, Denmark; Department of Physics and Astronomy, Aarhus University, Aarhus, Denmark; Center for Biomedical Imaging, Department of Radiology, New York University School of Medicine, New York, NY, United States; Center for Advanced Imaging Innovation and Research (CAI2R), Department of Radiology, New York University School of Medicine, New York, NY, United States

**Keywords:** diffusion, microstructure, gray matter, exchange, membrane integrity

## Abstract

Evaluating tissue microstructure and membrane integrity in the living human brain through diffusion water exchange imaging is challenging due to requirements for a high signal-to-noise ratio and short diffusion times dictated by relatively fast exchange processes. The goal of this work was to demonstrate the feasibility of*in vivo*imaging of tissue micro-geometries and water exchange within the brain gray matter using the state-of-the-art Connectome 2.0 scanner equipped with an ultra-high-performance gradient system (maximum gradient strength = 500 mT/m, maximum slew rate = 600 T/m/s). We performed diffusion MRI measurements in 15 healthy volunteers at multiple diffusion times (13–30 ms) and*b*-values up to 17.5 ms/μm^2^. The anisotropic Kärger model was applied to estimate the apparent exchange time between intra-neurite and extracellular water in gray matter. The estimated exchange time across the cortical ribbon was around (median ± interquartile range) 13 ± 8 ms on Connectome 2.0, substantially faster than that measured using an imaging protocol compatible with Connectome 1.0-alike systems on the same cohort. Our investigation suggested that the apparent exchange time estimation using a Connectome 1.0 compatible protocol was more prone to residual noise floor biases due to the small time-dependent signal contrasts across diffusion times when the exchange is fast (≤20 ms). Furthermore, spatial variation of exchange time was observed across the cortex, where the motor cortex, somatosensory cortex, and visual cortex exhibit longer apparent exchange times than other cortical regions. Non-linear fitting for the anisotropic Kärger model was accelerated 100 times using a GPU-based pipeline compared with the conventional CPU-based approach. This study highlighted the importance of the chosen diffusion times and measures to address Rician noise in diffusion MRI (dMRI) data, which can have a substantial impact on the estimated apparent exchange time and require extra attention when comparing the results between various hardware setups.

## 1 Introduction

Understanding the dynamics of water exchange within the human brain provides a key window into deciphering pathological mechanisms and assessing tissue integrity in aging and a variety of neurological and psychiatric disorders (Bai et al., 2018,^2019^). In neural tissues, water can passively traverse lipid bilayers or via aquaporins, specialized membrane protein channels that facilitate water movement between extracellular and intracellular compartments (Papadopoulos & Verkman, 2013). Disruptions in water homeostasis in conditions such as brain edema or ischemic stroke may alter membrane permeability between water compartments (King et al., 2004). Recent research has also highlighted the role of ATPase channels in actively co-transporting water for maintaining osmotic balance and supporting normal brain function (MacAulay, 2021;Springer et al., 2023a,2023b;Williamson et al., 2023). Characterizing membrane permeability may offer new insights into physiological processes and holds promise for disease monitoring and therapeutic intervention (Badaut et al., 2014). By elucidating these mechanisms, researchers can potentially identify novel targets for intervention and refine treatment strategies aimed at preserving brain health.

Diffusion MRI (dMRI) is a versatile imaging modality for investigating brain tissue microstructure noninvasively (Basser, 1997;Le Bihan, 1995). By harnessing magnetic field gradients, dMRI encodes the displacement of water molecules through the signal attenuation incurred as water molecules diffuse randomly and cause irreversible phase dispersion (Carr & Purcell, 1954;Stejskal & Tanner, 1965). In neural tissues, water diffusion is often restricted or hindered by cell membranes and organelles (Beaulieu, 2002). Understanding how diffusion-induced signal attenuation varies as a function of diffusion-encoding gradient field strength, duration, and diffusion times with respect to tissue microscopic structure promises to yield valuable histological-level information. One of the most widely applied uses of dMRI is the study of white matter microstructure (Assaf et al., 2004,^2008^;Fan et al., 2020;Fieremans et al., 2011;Huang et al., 2020;Jespersen et al., 2007;Kaden et al., 2016;Zhang et al., 2012). The prevailing biophysical model of diffusion in white matter involves a collection of fiber bundles (fascicles), each comprising aligned anisotropic water compartments with Gaussian diffusion, characterized by distinct diffusivities and water fractions, corresponding to the water inside and outside axons (Novikov, Veraart, et al., 2018;Novikov et al., 2019). Due to the presence of myelin sheath, water exchange between the compartments within a bundle is neglected over typically used diffusion times (Nilsson et al., 2013;Veraart et al., 2019,^2020^). The orientations of such multicompartmental fiber bundles within a voxel are characterized by a fiber orientation distribution function (ODF). This model, involving both the compartmental parameters of the bundle and the orientational parameters of the ODF, is commonly referred to as the Standard Model of diffusion in white matter (Novikov, Veraart, et al., 2018;Novikov et al., 2019).

Extending the Standard Model onto gray matter requires accounting for its specific microstructure properties. First, the soma compartment, corresponding to the cell bodies of neurons and glial cells, constitutes a significant portion of the gray matter volume (10–20%) (Shapson-Coe et al., 2024) and has a distinct isotropic and fully restricted contribution to the MRI signal (Palombo et al., 2020). Second, while myelin acts as a major barrier to diffusion and intercompartmental exchange in white matter, its concentration in gray matter is notably lower (Shapson-Coe et al., 2024), though both myelinated and unmyelinated axons can coexist in a voxel. The thin, branch-like cellular structures (neurites), such as unmyelinated axons, dendrites, and glial cell processes, may contribute to a non-negligible water exchange between neurites and extracellular space across the neurite membrane given the relatively high surface-area-to-volume ratio and/or other water transport mechanisms, such as via aquaporins and ATPase channels (MacAulay, 2021;Papadopoulos & Verkman, 2013;Springer et al., 2023a,2023b;Veraart et al., 2020;Williamson et al., 2023).

The presence of intercompartmental water exchange and restricted diffusion can be disentangled through multidimensional spectroscopy methods such as diffusion exchange spectroscopy (DEXSY) (Callaghan & Furó, 2004). In DEXSY, water exchange between environments with different diffusivities is observed during the mixing time of a double diffusion encoding scheme, manifesting as off-diagonal elements in the 2-dimensional spectrum (Callaghan & Furó, 2004). However, DEXSY demands a large amount of data to compute the multidimensional exchange spectrum, which limits its direct applicability in imaging contexts. Efforts have been made to shorten the lengthy acquisition of DEXSY (Åslund et al.,^2009^;Cai et al., 2018;Williamson et al., 2019,^2020^), notably through filtered exchange imaging (FEXI) (Lasič et al., 2011), a variant tailored for clinical feasibility. Nonetheless, the interpretation of apparent exchange rates derived from FEXI can be confounded by water exchange between the intra- and extra-vascular environments (Bai et al., 2020) and the geometry of the involved compartments (Khateri et al., 2022;Li et al., 2023). Despite the challenges facing FEXI, previous research underscores the association between apparent exchange rates and membrane permeability (Nilsson et al., 2013;Ning et al., 2018).

To shorten the lengthy multidimensional DEXSY-like experiments, tissue microstructure and intercompartmental water exchange can be assessed by employing a parsimonious*model*of exchange between just a few compartments. Models reduce the number of parameters making their estimation more robust and less SNR demanding, yet they rely on explicit assumptions for the microstructure (Novikov, Kiselev, et al., 2018). The most widespread model of exchange in the dMRI context has been suggested byKärger (1985). In its original formulation, exchange was postulated to occur between two isotropic compartments with otherwise Gaussian diffusion, at each point of the sample, governed by simple rate equations in the spirit ofZimmerman and Brittin (1957).

Extending the Kärger model (KM) onto structurally rich microstructure involved clarifying its assumptions. It was realized that KM applies only at sufficiently long diffusion times, after*coarse-graining*the tissue microstructure by the diffusion process, that is, at the scales exceeding cell sizes and the correlation length$l_{c}$of cell packing, assuming that the exchange time$t_{ex}\gg t_{c}$is also slow on the scale of the corresponding correlation time$t_{c}∼l_{c}^{2}/D$(Fieremans et al., 2010). In other words, KM is an*effective theory*valid at long times$t,\;t_{ex}\gg t_{c}$and small wave vectors$q\ll 1/l_{c}$, at which point the assumptions of Gaussian diffusion and exchange occurring at every point in space can become justified, and ordinary rate equations can be used instead of solving a genuine partial differential equation for the diffusion in a complex tissue geometry.

Compartment anisotropy, essential for neuronal tissue, can be further incorporated into KM (understood as an effective theory), using the steps analogous to building the Standard Model. First, exchange within a perfectly aligned fiber bundle (fascicle) of neurites and their extra-neurite (extracellular) space is added (assuming$t,\;t_{ex}\gg t_{c})$, yielding the*anisotropic Kärger model*for aligned fibers (Fieremans et al., 2010). Next, as in the Standard Model, the response of such an elementary bundle is convolved with an arbitrary fiber ODF, leading to the Neurite Exchange Imaging (NEXI) (Jelescu et al., 2022) and Standard Model with Exchange (SMEX) (Olesen et al., 2022) models suggested recently for gray matter, where the parameter constraint of isotropic extra-neurite space is further employed for the estimation robustness. We can thereby refer to NEXI/SMEX as the implementations of a*locally anisotropic*(or microscopically anisotropic) Kärger model, even if the overall signal from gray matter can be fairly isotropic due to an almost isotropic ODF.

Fitting such anisotropic extensions of the Kärger model, or its cumulants such as the time-dependent kurtosis (Fieremans et al., 2010), to the diffusion signals measured at multiple diffusion times enables the quantification of exchange time scales (Jelescu et al., 2022;Lee et al., 2020;Olesen et al., 2022). A prominent indicator showing the dominance of the exchange phenomenon is the relative decrease in the direction-averaged (spherical mean) signal as diffusion time increases for the same diffusion weighting, a pattern observed in recent investigations focusing on the gray matter of the mouse (Jelescu et al., 2022;Olesen et al., 2022) and human brain (Lee et al., 2022;Uhl et al., 2024) and in stoke (Lampinen et al., 2021;Lätt et al., 2009). The main advantage of the Kärger model is its straightforward implementation using the conventional pulsed-gradient dMRI sequence, despite the demands on gradient hardware to detect fast exchange effects (Williamson et al., 2019,^2023^) at short diffusion times. To facilitate efficient model fitting for exchange time evaluation, the narrow pulse approximation has been used to derive the analytical solution of the anisotropic Kärger model (Jelescu et al., 2022), though the potential impact of this approximation on exchange time measurements in clinically relevant scenarios with varying signal-to-noise ratios (SNR) remains unclear. Clarifying this aspect is crucial for the robust interpretation of exchange dynamics in clinical and preclinical settings.

Recent advances in MRI gradient systems present significant opportunities for*in vivo*tissue microstructure imaging using dMRI (Feinberg et al., 2023;Foo et al., 2020;Huang et al., 2021). The first-generation Connectome MRI scanner (Connectome 1.0; C1) was equipped with a high-performance gradient system achieving a maximum strength of 300 mT/m and a maximum slew rate of 200 T/m/s (Setsompop et al., 2013). This system enabled*in vivo*human dMRI with enhanced diffusion weighting and superior SNR compared with clinical scanners. Recent studies have demonstrated the feasibility of employing such a system to probe the*in vivo*exchange time using pulsed-gradient–based NEXI (Uhl et al., 2024) or dMRI of free gradient waveform (Chakwizira et al., 2023). Recently, a second-generation Connectome MRI scanner (Connectome 2.0; C2) was developed, featuring an improved high-performance gradient system capable of generating the strongest gradients for head-only*in vivo*human imaging at 3T to date, with a maximum gradient strength of 500 mT/m and a maximum slew rate of 600 T/m/s (Huang et al., 2021). These enhancements offer the potential for dMRI measurements at even stronger diffusion weighting with reasonable SNR, facilitating the exploration of intercompartmental exchange effects across a wider range of diffusion times using the Karger models.

The increasing availability of high-performance gradient systems for*in vivo*imaging, such as the Siemens Cima.X system (maximum strength of 200 mT/m, maximum slew rate of 200 T/m/s, whole-body 3T), GE SIGNA MAGNUS system (Foo et al., 2020) with HyperG gradients (maximum strength of 300 mT/m, maximum slew rate of 750 T/m/s, head-only 3T), and high-performance gradient insert (maximum strength of 200 mT/m, maximum slew rate of 600 T/m/s) (Weiger et al., 2018), provides essential feasibility to study the intercompartmental exchange times on*in vivo*human brain using NEXI. The primary goal of this study has been to investigate the apparent intercompartmental exchange times across the living human cortex using the stronger and faster gradients of the Connectome 2.0 MRI scanner compared with those estimated with a protocol compatible with the Connectome 1.0-alike systems using NEXI. Furthermore, we sought to evaluate the impact of the narrow pulse approximation on exchange time estimation. Finally, we demonstrated a novel tool leveraging GPU processing to accelerate the NEXI model fitting process, thereby enhancing the efficiency and scalability of parameter estimation for tissue microstructure imaging.

## 2 Theory

### 2.1 Anisotropic Kärger model

**Fig. 1.**
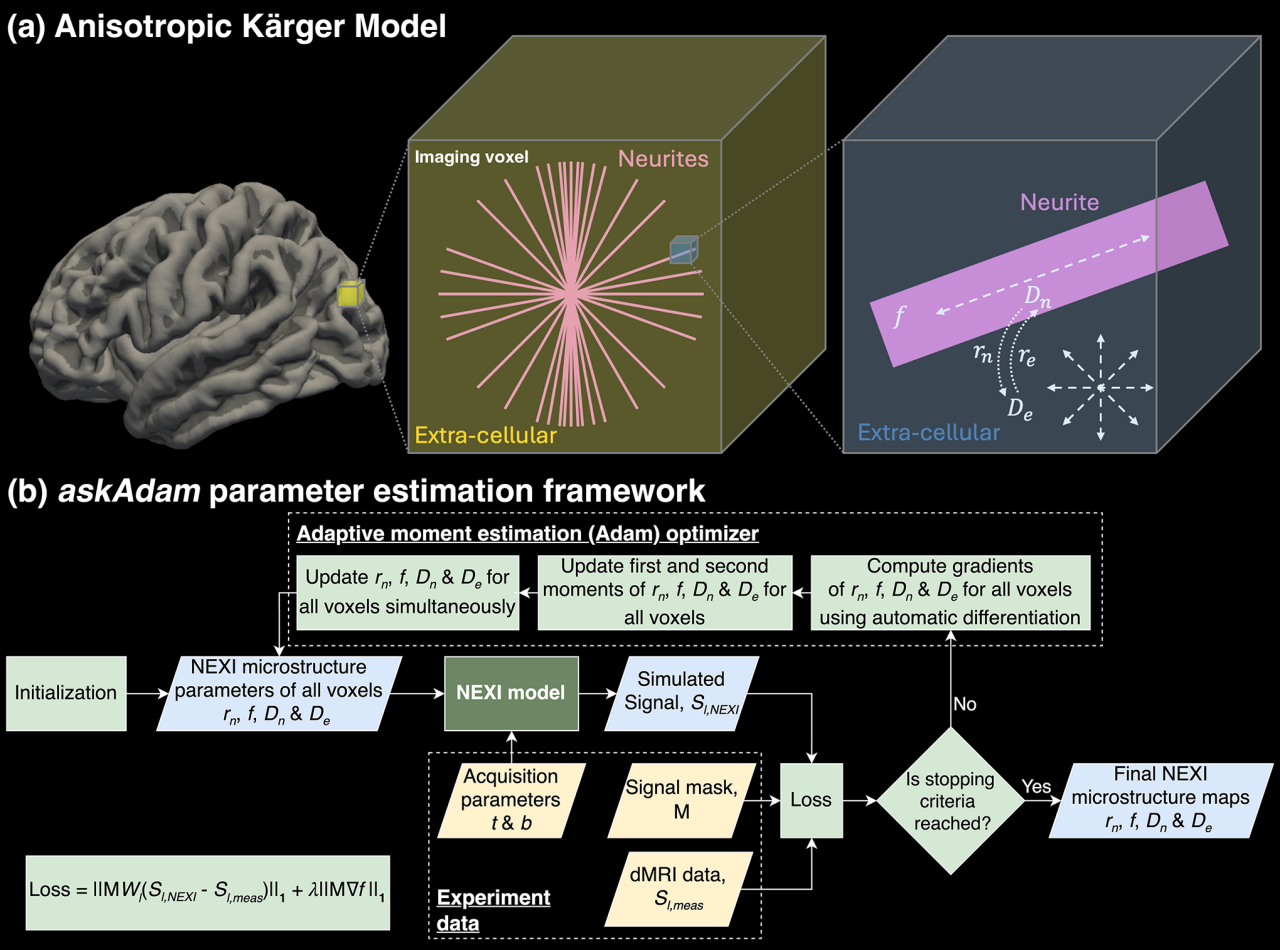
(a) Graphical illustration of the anisotropic Kärger model comprising the neurite compartment and extracellular water compartment. Each MRI voxel contains multiple neurites with various orientations. Intercompartmental water exchange takes place between neurites and extracellular water through various water movement mechanisms. (b) Illustration of the askAdam parameter estimation approach used in this work.

We utilized the anisotropic Kärger model (Fieremans et al., 2010;Jelescu et al., 2022;Olesen et al., 2022) to model the exchange effect between neurites and extracellular water in gray matter (Fig. 1a). Briefly, the anisotropic Kärger model comprises two exchanging Gaussian compartments: an anisotropic “stick”-like neurite compartment with zero diffusivity perpendicular to the long axis, and its local extracellular space. We assumed isotropic diffusion in the extracellular space to simplify the signal model and improve the robustness of estimation by reducing the number of model parameters. Considering a medium composed of neurites (n) and the extracellular water (e), its signal representation can be written as (Fieremans et al., 2010;Kärger, 1985;Ning et al., 2018):

$$\frac{d}{dt}\;\;\left[\begin{array}{c} M_{n}\left(t\right)\\ M_{e}\left(t\right) \end{array}\right]=\left(\left[\begin{array}{cc} -r_{n} & r_{e}\\ r_{n} & -r_{e} \end{array}\right]-q^{2}\left(t\right)\left[\begin{array}{cc} D_{n}\varepsilon^{2} & 0\\ 0 & D_{e} \end{array}\right]\right)\left[\begin{array}{c} M_{n}\left(t\right)\\ M_{e}\left(t\right) \end{array}\right],$$ (1)

where$M_{\left\{n,e\right\}}(t)$are both T_1_-weighted and T_2_-weighted magnetizations of neurite and extracellular water, respectively, at diffusion timet,$r_{\left\{n,e\right\}}$are the exchange rates from neurite to extracellular water and vice versa, such that the two compartments satisfy the detailed balance$r_{n}f_{n}=r_{e}f_{e}$with neurite volume fraction$f_{n}=f=\frac{M_{n}(0)}{M_{n}(0)+M_{e}(0)}$and extracellular volume fraction$f_{e}=1-f$, and$D_{\left\{n,e\right\}}$are the along-neurite and isotropic extracellular water diffusivities, respectively. Here, we assumed the longitudinal and transverse relaxation times of the neurite and extracellular water to be the same, and the echo time dependence due to transverse relaxation and the effect of coil sensitivity are cancelled out after normalizing the measurements by the*b*= 0 signal. The diffusion wave vectorq(t)represents the diffusion gradient profileG(t)such that$q(t)=\gamma \int_{0}^{t}G(t\prime )\text{d}t\prime$with the gyromagnetic ratioγand*b*-value$b=\int_{0}^{t}q^{2}(t^{\prime })\text{d}t^{\prime }$defining the diffusion weighting. The directional dependence is embodied in the dot product*$\varepsilon =\hat{n}\cdot \hat{g}$*between the neurite orientation$\hat{n}$and the diffusion gradient direction$\hat{g}$. The exchange time between the neurite and extracellular water is defined as$t_{ex}\;=1/(r_{n}+r_{e})\;=(1-f)/r_{n}$. To account for the fiber dispersion of neurites in an MRI voxel, the diffusion signal$S(b,\hat{g},t)$can be expressed as the convolution of the overall signal kernel$K\left(b,\varepsilon ,t\right)=M_{n}+M_{e}$fromEq. (1)with the ODF$P(\hat{n})$(Novikov, Veraart, et al., 2018):

$$S\left(b,\hat{g},t\right)=\int_{\left|\hat{n}\right|=1}\text{d}\hat{n}\;P(\hat{n})K\left(b,\varepsilon ,t\right).$$ (2)

For any linear tensor encoding gradient waveform, for example, pulsed-gradient sequence of finite pulse width (Stejskal & Tanner, 1965), the overall fiber responseK(b,ε,t)enteringEq. (2)can be calculated by numerically solving the system of coupled differential equationsEq. (1). Here, we refer to this method as SMEX (Olesen et al., 2022). It is worth noting that while the soma compartment is not explicitly accounted for in the NEXI/SMEX model, it is considered part of the extracellular compartment by assuming that the soma and extracellular water have similar diffusion properties and/or being well mixed (Olesen et al., 2022). Further, the exchange between soma and extracellular compartments is assumed negligible due to the small surface-to-volume ratio of somas, compared with neurites. More details about the theory and assumptions can be found inOlesen et al. (2022).

WhileEq. (1)is a general representation of the fiber bundle (fascicle) response for any gradient profile, computing the dMRI signals requires solving the coupled equations inEq. (1)using numerical solvers, which is computationally costly and results in a lengthy model fitting process. Alternatively, under the narrow pulse approximation, the solution ofEq. (1)has an analytical form (Fieremans et al., 2010;Kärger, 1985):

$$K\left(b,\varepsilon ,t\right)=f_{1}^{\prime }e^{-a_{1}}+f_{2}^{\prime }e^{-a_{2}},$$ (3)

a1,2=(12bDnε2+bDe+trn+tre   ±(bDnε2−bDe+trn−tre)2+4trnre), (4)

$$f_{1,2}^{\prime }\equiv \pm \frac{b(fD_{n}\varepsilon^{2}+(1-f)D_{e})-a_{2,1}}{a_{1}-a_{2}},$$ (5)

which has been referred to as NEXI (Jelescu et al., 2022).

The NEXI microstructure parameters,f,$D_{n}$,$D_{e},$and$r_{n}$, can be estimated using the rotationally invariant mapping framework (Novikov, Veraart, et al., 2018;Reisert et al., 2017) in which both the signal model and the measured dMRI signal are projected onto the spherical harmonics space after normalizing the diffusion-weighted signal by*b*= 0 signal. Thel-th order rotationally invariant signal$S_{l}$can be represented as

$$S_{l}(b,t,f,D_{n},D_{e},r_{n})=p_{l}K_{l}(b,t,f,D_{n},D_{e},r_{n}),l=0,2,\ldots$$ (6)

where the rotational invariant$p_{l}$characterizes the ODF anisotropy for each orderland$p_{0}\equiv 1$(ODF normalization), and$K_{l}$is the projection of signal kernelK(b,ε,t)onto the Legendre polynomials. Specifically, the zeroth-order signal invariant$S_{l\;=\;0}$corresponds to the commonly known directionally averaged, spherical mean signal (Kaden et al., 2016). Higher-order invariants$S_{l=2,4,\ldots }$may also contain microstructure information when the ODF is anisotropic. Note that the estimated values of the microstructure parameters in the kernel responseKare model specific and should be regarded as*apparent*tissue properties, as any modifications of the biophysical model and its assumptions would likely affect the estimated values.

### 2.2 GPU-accelerated “askAdam” framework for NEXI parameter estimation

Estimation of the NEXI microstructure parameters fromEqs. [3–6] involves optimization on a 4-dimensional parameter space (one more additional dimension for every orderlfroml=2,4,6,...). Utilizing conventional non-linear least-square approaches based on CPU computation for NEXI parameter estimation can be computationally lengthy for whole-brain mapping: the fitting process is repeated hundreds of thousands of times sequentially over all voxels in an imaging volume. Recent research has demonstrated the efficiency of accelerating the parameter estimation process based on GPU computation, both on minimization-based (Harms et al., 2017;Hernandez-Fernandez et al., 2019) and supervised learning-based approaches (Jung et al., 2021;J. Lee et al., 2019;Martins et al., 2021;Nedjati-Gilani et al., 2017). Here, we incorporated NEXI parameter estimation into the “askAdam” framework (Chan et al., 2022;Marques et al., 2023), one of the minimization-based GPU-accelerated methods, aiming to provide an additional resource to shorten the estimation processing time while maintaining similar estimation performance to the conventional CPU-based non-linear least-square (NLLS) approach.

“AskAdam” is a parameter estimation tool (Chan et al., 2022;Marques et al., 2023), leveraging the efficiency and suitability of the stochastic gradient descent-based optimizer, such as Adam, to handle large non-convex problems. The Adam optimizer is commonly used in deep learning to perform learnable network parameter updates (Kingma & Ba, 2014) (Fig. 1b). To enable askAdam to process multiple voxels at the same time in NEXI parameter mapping, the optimization problem can be formulated as:

$${\text{argmin}}_{f,D_{n},D_{e},r_{n}}{\left\|\;\text{M}W_{l}(S_{l,meas}(b,t)-S_{l}(b,t,f,\;D_{n},D_{e},r_{n}))\;\right\|}_{\text{L}1/\text{L}2/\text{MSE}},$$ (7)

whereMis the (brain) signal mask,$W_{l}=1/\sqrt{4\pi (2l+1)}$is the weight associated with thel-th order rotationally invariant signal (Novikov, Veraart, et al., 2018), and$S_{l,meas}$is thel-th order rotationally invariant signal from the actual measurements. The optimization loss shown inEq. (7)is nearly identical to that used for an NLLS solver in conventional voxel-wise fitting. The main difference is that all input voxels inside the signal mask (which can be a full set or subset of the whole-brain data, depending on the memory allowance of the GPU) contribute to one loss in a single optimization problem in the askAdam framework. Here, the initialization of the NEXI microstructure parameters can be done by using random values or prior estimates as in NLLS. During each optimization cycle, the NEXI microstructure parameters of all voxels are incorporated with the sequence settings (e.g., diffusion times and*b*-values) to forward simulate the NEXI signals across the entire volume using element-wise arithmetic operations to derive the loss inEq. (7). The loss gradient with respect to the model parameters on each voxel is then computed using the automatic differentiation function from Matlab, so that the model parameter across the entire dataset can be updated simultaneously using the Adam optimizer (Fig. 1b). All computations (i.e., forward signal generation, gradient computation, and parameter update) can take place on a GPU to accelerate the optimization process. This process is repeated until the stopping criteria are fulfilled (either the maximum number of optimization iterations is reached or the change of loss across iterations is less than a certain tolerance value; a maximum of 4000 iterations and a tolerance level of 1 x 10^-8^were chosen here). We choose the L1-norm as the loss function inEq. (7)as it outperforms the L2-norm at moderate SNR in our preliminary analysis (seeSupplementary Fig. S1).

Another advantage of askAdam is that the loss function can be further customized to include spatial regularization as part of the optimization problem, which has been used in image reconstruction (Knoll et al., 2011), quantitative susceptibility mapping (QSM Consensus Organization Committee et al., 2024), and multi-compartment parameter fitting (Orton et al., 2014;Pasternak et al., 2008) to improve the estimation results. To do so, the loss function ofEq. (7)can be generalized into

$${\text{argmin}}_{f,D_{n},D_{e},r_{n}}{\left\|\;\text{M}W_{l}(S_{l,meas}(b,t)-S_{l}(b,t,f,D_{n},D_{e},r_{n}))\;\right\|}_{\text{L}1/\text{L}2/\text{MSE}}+\;\lambda ℛ,$$ (8)

whereλis a regularization parameter andℛcan be any regularizer that promotes certain features (for example, sparsity or smoothness) in the estimation maps. This feature is feasible for askAdam because the microstructure parameters of all voxels update simultaneously during the model fitting process, in turn, allowing the regularizer to stay up to date during the optimization cycle.

## 3 Methods

### 3.1 Numerical simulations

#### 3.1.1 Noise propagation with forward SMEX and NEXI signal generation

To address the research question of whether the narrow pulse solution employed in NEXI is sufficient to represent the dMRI signal in an actual experiment for high-gradient performance MRI systems, we conducted a noise propagation analysis to evaluate the estimation performance of the NEXI model fitting using SMEX and NEXI for forward signal generation with realistic dMRI acquisition protocols designed for either the Connectome 1.0 or Connectome 2.0 scanner as shown inTable 1. We randomly chose 10,000 different combinations of the NEXI microstructure parameters within the following range:$t_{ex}$ = [1, 50] ms,f = [0.01, 0.99],$D_{n}$ = [0.1, 3] μm^2^/ms, and$D_{e}$ = [0.1, 3] μm^2^/ms with$D_{n}$ ≥$D_{e}$. Complex-valued Gaussian noise was applied to the simulated signals to simulate a clinically relevant SNR of 50 in the non-diffusion-weighted*b*= 0 signal. Realistic Rician noise conditions were simulated by extracting the magnitude of the signal with complex-valued Gaussian noise. NEXI model fitting was subsequently performed on the spherical mean (zeroth-order rotational invariant) after normalized to*b*= 0 images. The starting point of the estimation for each voxel was set up using a dictionary-matching approach (Ma et al., 2013), achieved by searching the maximum likelihood of the inner product of the*in vivo*measured signal to a dictionary of 10,000 randomly generated simulated signal across a predefined range of$r_{n}$ = [0.001, 0.99] s^-1^,f = [0.01, 0.99],$D_{n}$ = [1.5, 3] μm^2^/ms and$D_{e}$ = [0.5, 1.5] μm^2^/ms. The fitting boundary of each NEXI parameter was set to$r_{n}$ = [0, 1] s^-1^,f = [0, 1],$D_{n}$ = [0.1, 3] μm^2^/ms,$D_{e}$ = [0.1, 3] μm^2^/ms, and we did not impose the same constrain of$D_{n}$ ≥$D_{e}$as in the ground truth used in forward signal simulation. The forward SMEX and NEXI signals were calculated for 64 diffusion gradient directions per shell. Additionally, we investigated a noise-floor-correction method for the signal with Rician noise by integrating the Rician noise floor within the signal model by using the Rician mean model (Jelescu et al., 2022). We repeated this process based on the Connectome 2.0 protocol with varying diffusion gradient pulse durationδ=8 ms and 10 ms, respectively, to consider its impact on Connectome 1.0-alike systems with a relatively lower maximum gradient amplitude and slower gradient slew rate.

**Table 1.** Data acquisition protocols for dMRI in this study.

Protocol	Connectome 2.0			Connectome 1.0		
Diffusion time t (ms)	13	21	30	21	30	40
Diffusion gradient duration δ (ms)	6			10		
# gradient directions per shell	32 (for C1 vs. C2 comparison)/64 (for high SNR cortical investigation)			32		
# shells	5	6	7	5	6	7
*b* -values (ms/μm ^2^ )	[1*,2.3,3.5,4.8,6.5]	[1*,2.3,3.5,4.8,6.5,11.5]	[1*,2.3,3.5,4.8,6.5,11.5,17.5]	[1*,2.3,3.5,4.8,6.5]	[1*,2.3,3.5,4.8,6.5,11.5]	[1*,2.3,3.5,4.8,6.5,11.5,17.5]
T _acq_ (min)	21	25	29.5	24	29	34

The same protocol parameters are used in the noise propagation analysis and*in vivo*imaging. Diffusion gradient duration is defined as the full width at half maximum of the gradient pulse. The maximum ramp time of the gradient pulse was (500/600=)0.83 ms for the Connectome 2.0 protocol and (300/200=)1.5 ms for the Connectome 1.0 protocol. For each diffusion time, the gradient strength was the only control variable to achieve various*b*-values, and the highest*b*-value utilized the maximum gradient strength of 300 mT/m on the Connectome 1.0 protocol and 500 mT/m on the Connectome 2.0 protocol. *b= 1 ms/μm^2^data were not used in data fitting.

#### 3.1.2 Noise propagation with ${\text{l}}_{\text{max}}=0$ and ${\text{l}}_{\text{max}}=2$ NEXI models

As introduced in the Theory section, NEXI model fitting is not limited to the spherical mean signal (zeroth-order rotational invariant), but can involve any spherical harmonics order up to$l_{max}$, depending on the total number of unique gradient directions acquired for each*b*-value. In the second noise propagation analysis, we investigated whether the estimation performance could be improved when the second-order rotational invariants of the dMRI signals were added to the model fitting. From now on, we refer to fitting the zeroth-order rotational invariant$S_{l\;=\;0}$of NEXI as$l_{max}\;=0$, and to fitting both invariants ($S_{l\;=\;0}$+$S_{l\;=\;2}$) of NEXI as$l_{max}=2$. Noise propagation was conducted under the same conditions as inSection 3.1.1, and the range of the non-linear neurite dispersion index$p_{2}$was within [0, 0.3] for the forward simulations. The$p_{2}$value increases with the tissue anisotropy (Novikov, Veraart, et al., 2018). The low$p_{2}$value in simulations was consistent with the low anisotropy observed in gray matter.

#### 3.1.3 Comparison between NLLS and askAdam on NEXI parameter mapping

The askAdam framework allows data fitting to be performed simultaneously in multiple spatial dimensions with the option to incorporate spatial regularization, which is distinct from voxel-wise NLLS fitting. Therefore, we wanted to understand how this spatial regularization would affect the estimation performance between the two solvers via an*in silico*head phantom experiment. The phantom was created using the*in vivo*NEXI fitting results of one participant scanned with the Connectome 2.0 scanner (see details inSection 3.2.4). The median value across each brain parcellation label (excluded ventricles and cerebrospinal fluid) was computed for all NEXI microstructure parameters and used as the ground truth for the corresponding region of interest (ROI) segmented using FreeSurfer (Section 3.2.2). We then forward simulated the NEXI signals using the Connectome 2.0 dMRI acquisition protocol inTable 1. Gaussian noise was added to the simulated signals so that we could investigate the estimation performance at a moderate SNR of 50 and a low SNR of 25. Tissue microstructure parametric maps were derived using the NEXI$l_{max}\;=2$model implementation via either NLLS or askAdam solvers. Additionally, we incorporated anisotropic, 2-dimensional spatial total variation (TV) regularization on the neurite fraction map for the NEXI model fitting with askAdam (askAdam_TV_). This was achieved by applying a 2D gradient operator ($\nabla =[\nabla_{\text{x}};\nabla_{\text{y}}]$) on thefmap in the in-plane direction, that is,$ℛ={\left\|\;\text{M}\nabla f\;\right\|}_{1}$inEq. (8)(Fig. 1b). The regularization parameterλis empirically set to 0.002 mm (seeSupplementary Fig. S2for details). We evaluated the accuracy of estimation via the median differences (bias) between the estimated values and ground truth, and the precision via the interquartile range (IQR) of the fitted values within each ROI. For all data fitting, the NLLS approach was performed on an EPYC 7313 16-Core Processor (AMD, Santa Clara, US), whereas the askAdam approach was performed on an A40 GPU (NVIDIA, Santa Clara, USA).

### 3.2 *In vivo* MRI

#### 3.2.1 Data acquisition

##### 3.2.1.1 Comparison between Connectome 1.0 and Connectome 2.0 protocols

Data acquisition was performed on the 3T Connectome 2.0 scanner (Ramos-Llordén et al., in press) (MAGNETOM Connectom.X, Siemens Healthineers, Erlangen, Germany) equipped with a maximum gradient strength of 500 mT/m and slew rate up to 600 T/m/s. Imaging experiments were performed on 5 healthy volunteers (mean ± SD age = 26 ± 6 years; 3 females, 2 males) using a custom-built 72-channel head coil for signal reception (Mahmutovic et al., 2024). The study was approved by the local ethics committee, and written informed consent was obtained from all participants for being included in the study. The imaging protocol comprised:

(1) Whole-brain T_1_-weighted scan using MPRAGE with 0.9 mm isotropic resolution, acquisition time = 5.5 min;
(2) 2D spin-echo EPI-dMRI, multi-band factor of 2, GRAPPA factor of 2, partial Fourier of 6/8, resolution = 2 mm isotropic, TR/TE_C1_/TE_C2_= 3600/68/54 ms using the diffusion scheme laid out inTable 1. Non-diffusion-weighted images (*b*= 0) were acquired interspersed for every 16 diffusion-weighted images (DWIs). Total acquisition time = 40 min per protocol.

##### 3.2.1.2 NEXI measurements with higher SNR data

Additionally, 10 healthy volunteers (mean ± SD age = 31 ± 15 years; 6 females, 4 males) were scanned using the same hardware and imaging sequences to measure the NEXI’s apparent exchange time with higher SNR data. The same C2 protocol as described inTable 1was used for this dataset, with the only modification being that 64 diffusion encoding directions were acquired for each*b*-value instead of 32. The total acquisition time is 80 min.

#### 3.2.2 Data processing

The MPRAGE images were processed using FreeSurfer’s*“recon-all”*function for cortical surface reconstruction (Fischl, 2012) and SynthSeg (Billot et al., 2023) for gray matter parcellation. Diffusion MRI data were processed based on a modified DESIGNER pipeline (Ades-Aron et al., 2018), including Rician-noise-floor-corrected Marchenko–Pastur Principal Component Analysis (MP-PCA) image denoising with three iterations (Tournier et al., 2023;Veraart et al., 2016), Gibbs ringing artifact removal on non-diffusion-weighted images based on local sub-voxel shifts in MRtrix (Kellner et al., 2016;Tournier et al., 2019), susceptibility-induced image distortion using FSL’s*topup*(Andersson et al., 2003), eddy current-induced distortion using FSL’s*eddy*(Andersson & Sotiropoulos, 2016), and gradient non-linearity distortion correction with in-house code (Fan et al., 2016;Jovicich et al., 2006). Image registration between MPRAGE and dMRI data was performed based on non-linear registration using ANTs (Avants et al., 2011). The transformation matrices and warp fields were subsequently used to transform MPRAGE-derived gray matter parcellation labels to dMRI space for statistical analysis and dMRI-derived parameter maps to MPRAGE space for visualization.

#### 3.2.3 Investigating time dependence via ROI fitting

We evaluated the time dependence of the*in vivo*dMRI signals in various areas of the gray matter on ROI-averaged diffusion signals. Six ROIs, the amygdala, hippocampus, frontal, parietal, temporal, and occipital gray matter, were created by combining the parcellation labels from SynthSeg according toKlein & Tourville (2012). Zeroth- and second-order rotationally invariant dMRI signals ($S_{l=\;0}$and$S_{l\;=\;2}$) on each voxel were derived by projecting the directionally dependent dMRI signal up to the fourth-order spherical harmonic after normalizing the DWIs by the mean*b*= 0 images ($S_{l=\;4}$was not used). The rotationally invariant signals were then averaged over each ROI and across all subjects. The averaged signals were fitted to both zeroth- and second-order ($l_{max}\;=2$) spherical harmonic of the NEXI model using the NLLS implementation. All diffusion times and*b*-values were used in the model fitting except data acquired at*b*= 1 ms/μm^2^since the restricted diffusion inside the soma compartment could still have non-negligible contributions to the dMRI signal at low*b*-values (seeSupplementary Fig. S6).

#### 3.2.4 NEXI microstructure parameter mapping

Tissue microstructure parametric maps were derived using the NEXI$l_{max}\;=2$model implementation on the whole-brain data for individual subjects via either the NLLS or askAdam solvers similar to the*in silico*head phantom experiment inSection 3.1.3. Additionally, we incorporated 2D-TV regularization for the NEXI model fitting with askAdam to validate the*in silico*experiment findings with the*in vivo*data. Subsequently, the apparent exchange time maps were transformed into FreeSurfer’s fsaverage subject space and projected onto the cortical surface for visualization.

#### 3.2.5 Statistical analysis

In subsequent statistical analyses on the whole-brain parameter maps, the median value within each cortical ROI was computed to minimize outliers due to potential partial volume effects and imperfect cortical surface registration between the MPRAGE and dMRI images for all the NEXI microstructure parameters on each subject. Group-level statistics were derived by computing the mean exchange time and its standard deviation (SD) across subjects for each ROI. With the 2 mm isotropic resolution of the*in vivo*data, the partial volume effect originating from the cerebrospinal fluid (CSF) and superficial white matter may affect the accuracy of the estimated gray matter exchange time. Therefore, we performed a Pearson’s correlation analysis to investigate whether the exchange time derived from NEXI was associated with the cortical thickness estimated from the SynthSeg parcellation result on all ROIs. Additionally, we computed the Pearson’s correlation coefficients between the NEXI microstructure parameters to understand how they interacted with one another.

## 4 Results

### 4.1 Noise propagation analysis

**Fig. 2.**
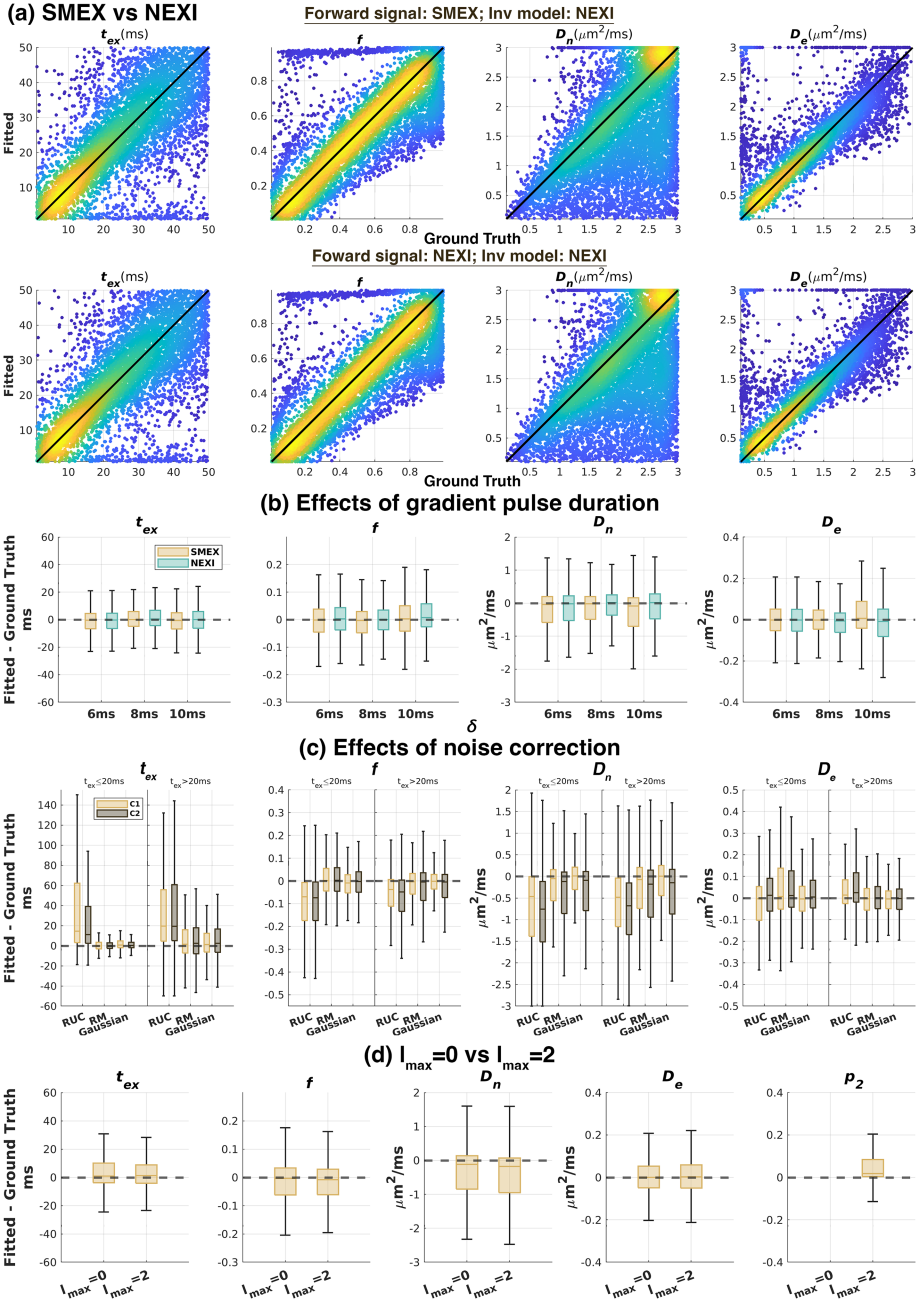
Noise propagation analysis evaluating the effect of the narrow pulse approximation for NEXI at SNR of 50. (a) Scatter plots of the estimated NEXI microstructure parameters versus ground-truth values on data with Gaussian noise using Connectome 2.0 protocol. Color scale corresponds to the occurrence on the spatial grid (Yellow: high occurrence; Blue: low occurrence). (b) Box plots of the NEXI parameters estimated with various gradient pulse durations. (c) Box plots of the NEXI parameters estimated with various Rician noise floor correction methods. (d) Box plots of the NEXI parameters estimated with l_max_= 0 and l_max_= 2.

Noise propagation results from fitting the zeroth-order rotational invariant of NEXI to either SMEX or NEXI for forward signal generation showed similar estimation performance on all parameters at an SNR of 50 based on Connectome 2.0 dMRI acquisition protocol (Fig. 2a). Similar observations can be made when the gradient pulse duration was set to 8 and 10 ms (Fig. 2b). We compared the three noise correction scenarios: Rician uncorrected noise (RUC), Rician mean model (RM), and Gaussian noise. Among the three cases, the uncorrected Rician noise signal had the worst performance with greater biases and wider IQRs for all parameters (Fig. 2c). Incorporating the Rician mean into the NEXI signal model (RM) or with the Gaussian noise greatly reduces the estimation biases and IQRs for all fitted parameters. Notably, the exchange time estimation from the Connectome 1.0 protocol is less accurate and less precise than those derived from Connectome 2.0 at faster exchange times (≤20 ms) in the presence of the Rician noise floor. In the relatively slower exchange time regime (>20 ms), the estimation performance between the C1 and C2 is comparable in all cases (left panel,Fig. 2c). Comparable noise performance was observed when using only the zeroth-order rotational invariant of the signal and incorporating the second-order rotational invariant into the NEXI model fitting, for all estimated parameters (Fig. 2d). The noise propagation analysis result shows that the additional fitting parameter$p_{2}$(corresponding to the non-linear fiber dispersion index) can be robustly measured without degrading the estimation performance of the other parameters (Fig. 2d).

### 4.2 NLLS versus askAdam

**Fig. 3.**
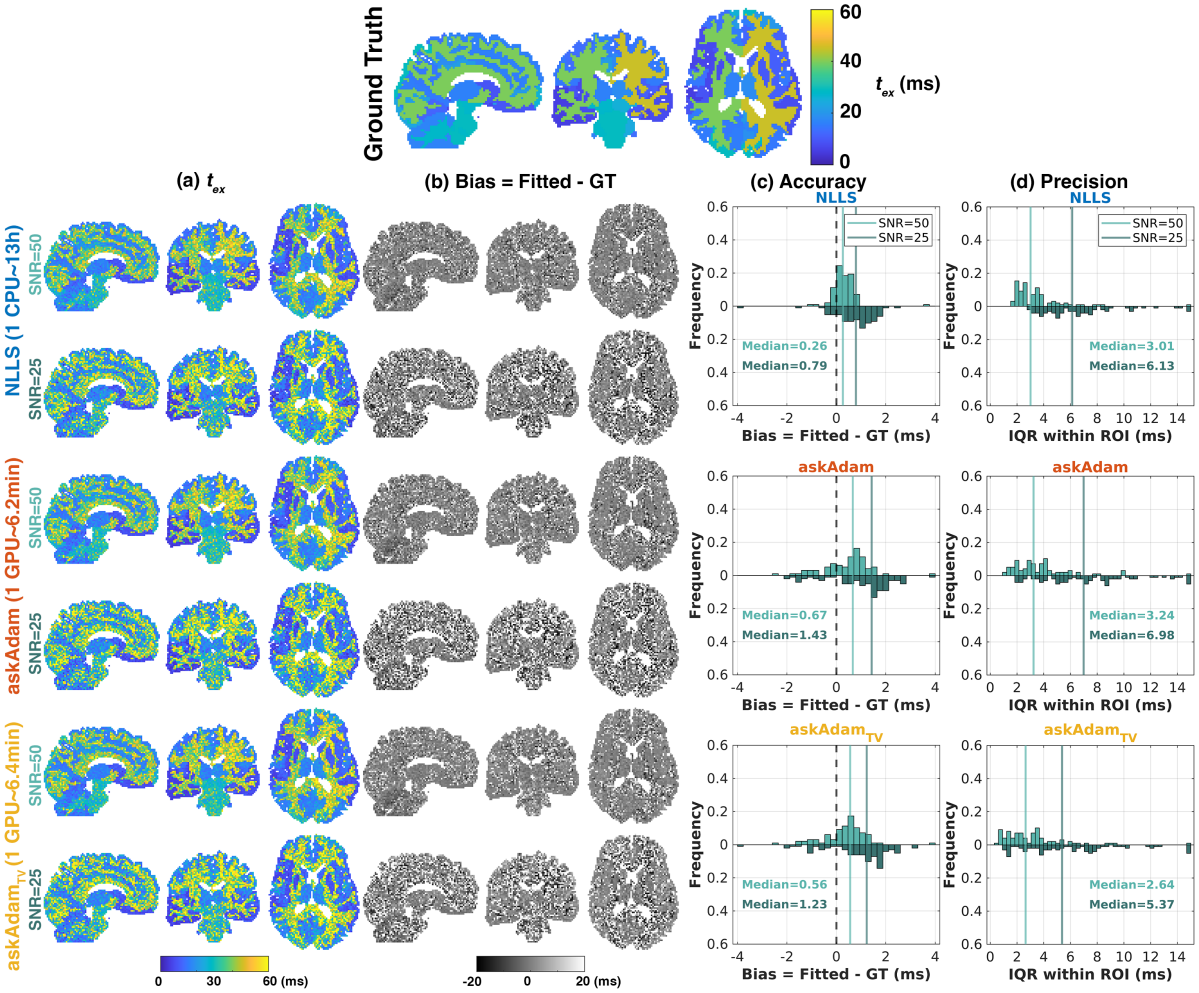
*In silico*head phantom comparison between NLLS and askAdam for NEXI parameter estimation performance. (a) The intercompartmental exchange time maps and (b) the corresponding estimation bias derived at SNR of 50 and SNR of 25 for the three solvers: voxel-wise NLLS, askAdam without spatial regularization, and askAdam with spatial regularization, (c) estimation accuracy, and (d) precision across all ROIs.

To determine whether askAdam can be an effective optimization tool for NEXI model fitting, we compared its estimation performance with the standard NLLS approach based on the summary statistics across ROIs using image-based numerical simulations. The processing time on the simulated whole-brain dataset phantom for the NLLS approach was about 13 h on 1 CPU, whereas the askAdam-based approaches took about 6 min, achieving over 100-fold acceleration on processing time by GPU. In terms of accuracy and precision, we observed that askAdam was slightly less accurate than NLLS for the exchange time estimation (median bias of NLLS = 0.26 ms vs. askAdam = 0.67 ms at SNR = 50;Fig. 3b, c), while the precision is comparable (median IQR of NLLS = 3.01 ms vs. askAdam = 3.24 ms,Fig. 3d). Interestingly, the measurement accuracy remains comparable with precision being improved when we introduced a weak spatial regularization with askAdam in NEXI model fitting (median bias of askAdam = 0.67 ms vs. askAdam_TV_= 0.56 ms; median IQR of askAdam = 3.24 ms vs. askAdam_TV_= 2.64 ms at SNR = 50). As SNR decreases, both the accuracy and precision of the estimation are reduced for all solvers (Fig. 3c, d).

### 4.3 *In vivo* results

**Fig. 4.**
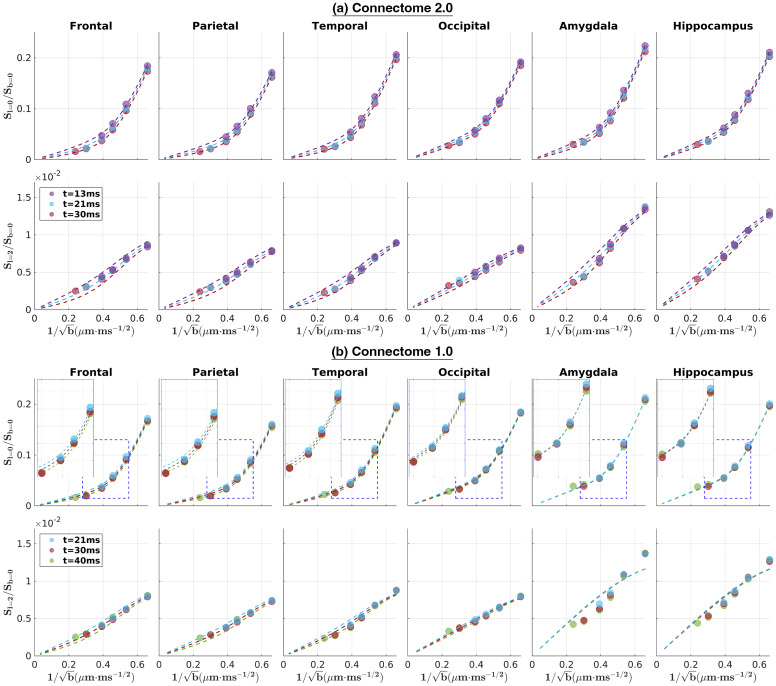
Results of ordinary NEXI model fitting on ROI-averaged*in vivo*dMRI signals. The NEXI model of$l_{max}\;=2$was fitted to data using the (a) Connectome 2.0 and (b) Connectome 1.0 protocol data. Circles represent the measured data and dashed lines represent the NEXI model fitting curves derived from the estimated tissue parameters.

We evaluated the goodness of fit between the NEXI model fitting and the*in vivo*data using the ROI-averaged rotationally invariant signals. Time-dependent dMRI signals were observed for all gray matter ROIs (circles inFig. 4) on both Connectome 2.0 (Fig. 4a) and Connectome 1.0 (Fig. 4b) protocol data, where both the zeroth- and second-order rotational invariants$S_{l=\;0}$and$S_{l=\;2}$decreased with diffusion time at each*b*-value. In terms of fit quality, the discrepancies between the fitted signal curves and the actual data were more noticeable on the second-order rotational invariants$S_{l=\;2}$, with the amygdala showing the poorest fit in both cases, though the root mean square values of the fitting residual are comparable between$S_{l=\;0}$and$S_{l=\;2}$, ranging from 2.26×10^-3^to 3.12×10^-3^for$S_{l=\;0}$and from 0.25×10^-3^to 1.41×10^-3^for$S_{l=\;2}$across ROIs. In this analysis, the estimated apparent$t_{ex}$ranged from 14.93 ms (frontal) to 33.51 ms (occipital) on the Connectome 2.0 data (Table 2), whereas the estimated$t_{ex}$from the Connectome 1.0 protocol was substantially longer than that from Connectome 2.0, though similar regional trends could be observed (faster exchange in the frontal, parietal, and temporal gray matter).

**Table 2.** Ordinary NEXI microstructure parameters of gray matter ROI derived from ROI-averaged*in vivo*dMRI signals.

	$t_{ex}$ (ms)		f		$D_{n}$ (μm ^2^ /ms)		$D_{e}$ (μm ^2^ /ms)		$p_{2}$	
	C2	C1	C2	C1	C2	C1	C2	C1	C2	C1
Frontal	14.93	30.22	0.35	0.27	3.00	3.00	0.89	0.90	0.21	0.24
Parietal	17.84	32.15	0.32	0.27	3.00	3.00	0.94	0.95	0.21	0.23
Temporal	17.06	39.39	0.35	0.28	3.00	3.00	0.81	0.81	0.20	0.23
Occipital	33.51	77.64	0.34	0.30	3.00	3.00	0.89	0.88	0.19	0.21
Amygdala	22.96	605.48	0.37	0.27	3.00	3.00	0.77	0.79	0.28	0.33
Hippocampus	29.36	323.18	0.36	0.28	3.00	3.00	0.82	0.83	0.28	0.32

C2: Connectome 2.0 protocol; C1: Connectome 1.0 protocol.

**Fig. 5.**
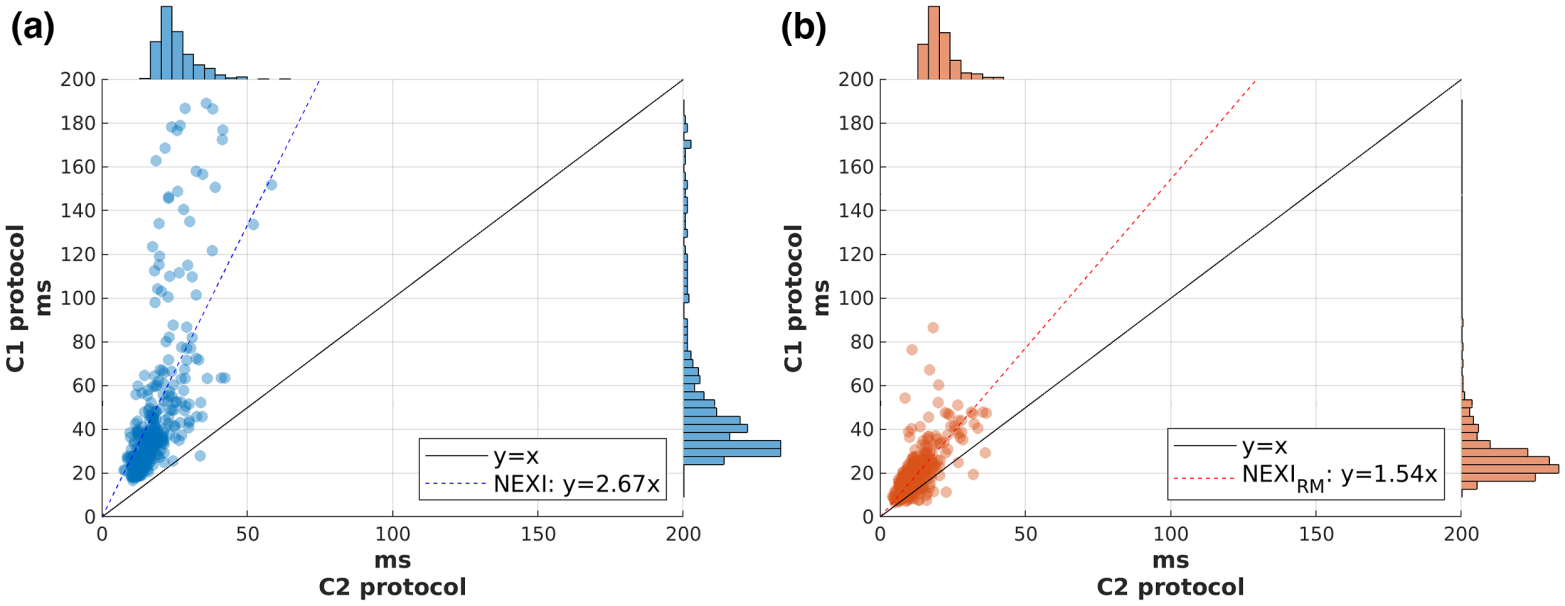
Scatterplots of the estimated exchange time using C1 and C2 protocols with (a) the ordinary NEXI model with denoise dMRI data and (b) the NEXI with Rician mean model with dMRI data that did not incorporate denoising in the preprocessing. Each data point corresponds to the median value of an ROI.

The*in vivo*whole-brain mapping estimation differences between the Connectome 2.0 and Connectome 1.0 protocols show similar patterns as fitting the ROI-averaged signal. The estimated$t_{ex}$with the Connectome 1.0 protocol was about 2.67 times longer than those with the Connectome 2.0 protocol when fitting the ordinary NEXI model to the denoise dMRI data (Fig. 5). Interestingly, the bias between the two protocols was reduced to a factor of 1.54 using the NEXI Rician mean model with no denoising applied to the dMRI data. For the Connectome 1.0 protocol, the apparent$t_{ex}$estimation of using NEXI Rician mean model was substantially shorter than those of using the ordinary NEXI model, while the$t_{ex}$estimations of the Connectome 2.0 protocol are comparable between the two approaches.

**Fig. 6.**
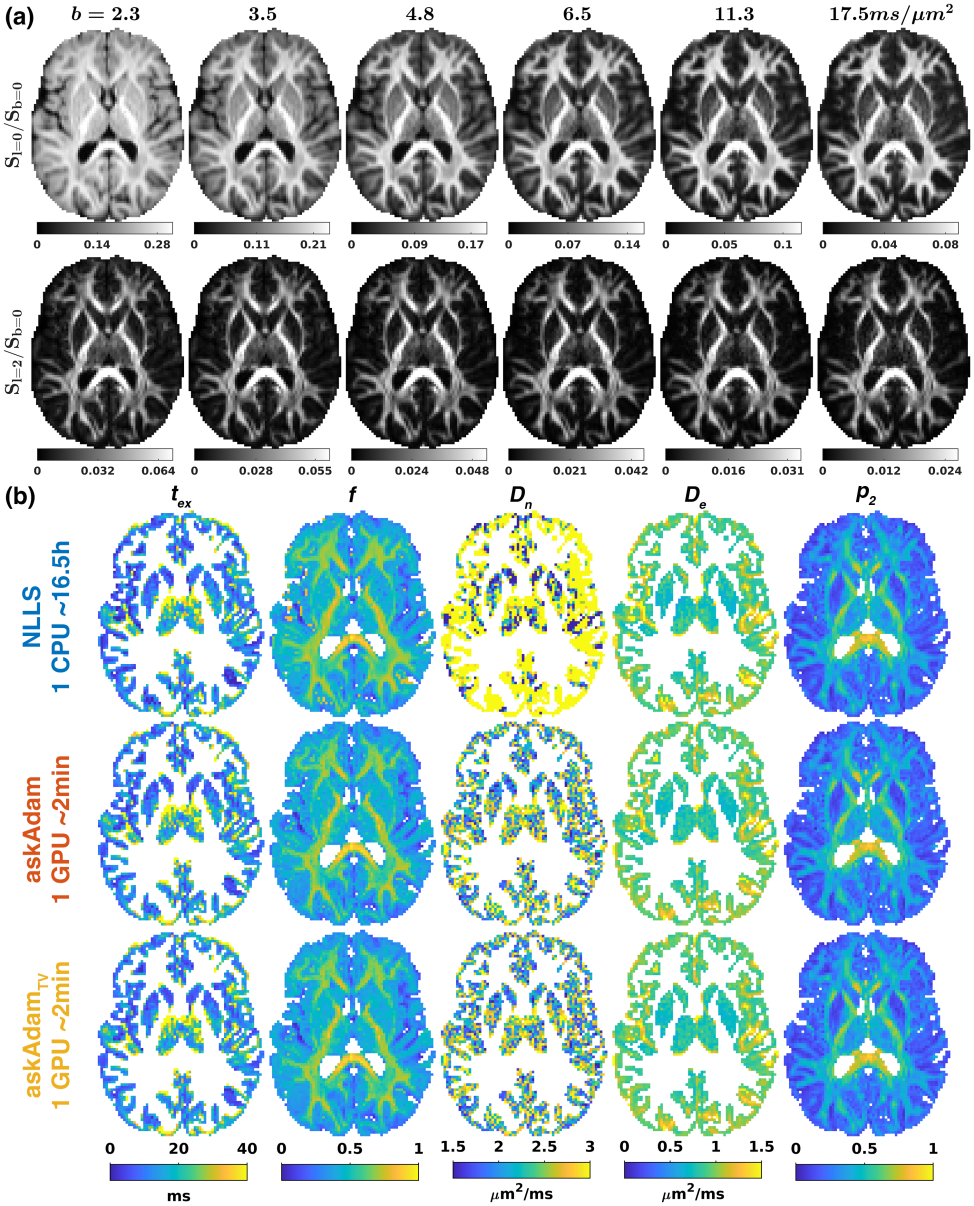
(a) Examples of the zeroth- and second-order rotationally invariant dMRI signals at*t*= 30 ms and*b*-values = 2.3–17.5 ms/µm^2^on one subject from the high SNR Connectome 2.0 cohort. Zoom-in images can be found inSupplementary Figure S8. (b) NEXI microstructure parameters on the same subject derived from different solvers. CSF (f and p_2_) and white matter (t_ex_, D_n_, and D_e_) were masked out to provide a better illustration of the estimations on gray matter.

Images of both the zeroth-order and second-order rotational invariants of the dMRI signal demonstrated that the Connectome 2.0 scanner produced very high-quality data even at high*b*-values (Fig. 6a). The mean SNR across all subjects in the cortex was 55 at*b*= 0 without data denoising. The SNR of the spherical mean signal (spherical mean signal divided by the noise estimation from*b*= 0 images) at*t*= 30 ms for each*b*-values from 2.3 to 17.5 ms/µm^2^was 9.6, 5.4, 3.4, 2.4, 1.7, and 1.5, respectively. These levels can be interpreted as the ratio of spherical mean signal-to-the-noise floor for each b-shell. On top of that, the noise fluctuation in spherical mean signal was even smaller by a factor of$\sqrt{N}$withN= 64 gradient directions per b-shell. The*in vivo*NEXI parameter maps on a single subject are shown inFigure 6b. Among all NEXI parameters, the apparent intra-neurite diffusivity$D_{n}$was the noisiest measurement. The estimated values were very close to the upper bound of the parameter range (3 μm^2^/ms), especially in white matter, whereas the estimations of neurite volume fractionfand ODF$p_{2}$were the most robust. All three optimization solvers (NLLS, askAdam, and askAdam_TV_) produced similar contrast on all parameters except$D_{n}$, where the values from askAdam were lower than those from NLLS and close to 2.5 μm^2^/ms.

The exchange time maps projected onto the cortical surface of two subjects (Subject #1: 23-year-old female; Subject #2: 22-year-old male) are shown inFigure 7. In both cases, the apparent$t_{ex}$maps derived from NLLS were apparently noisier than those from askAdam, and the results were comparable between with and without spatial regularization for askAdam. In these two examples, both subjects showed similar spatial contrasts between the pre-/post-central gyri, visual cortex, and cingulate gyrus to their surrounding tissue.

**Fig. 7.**
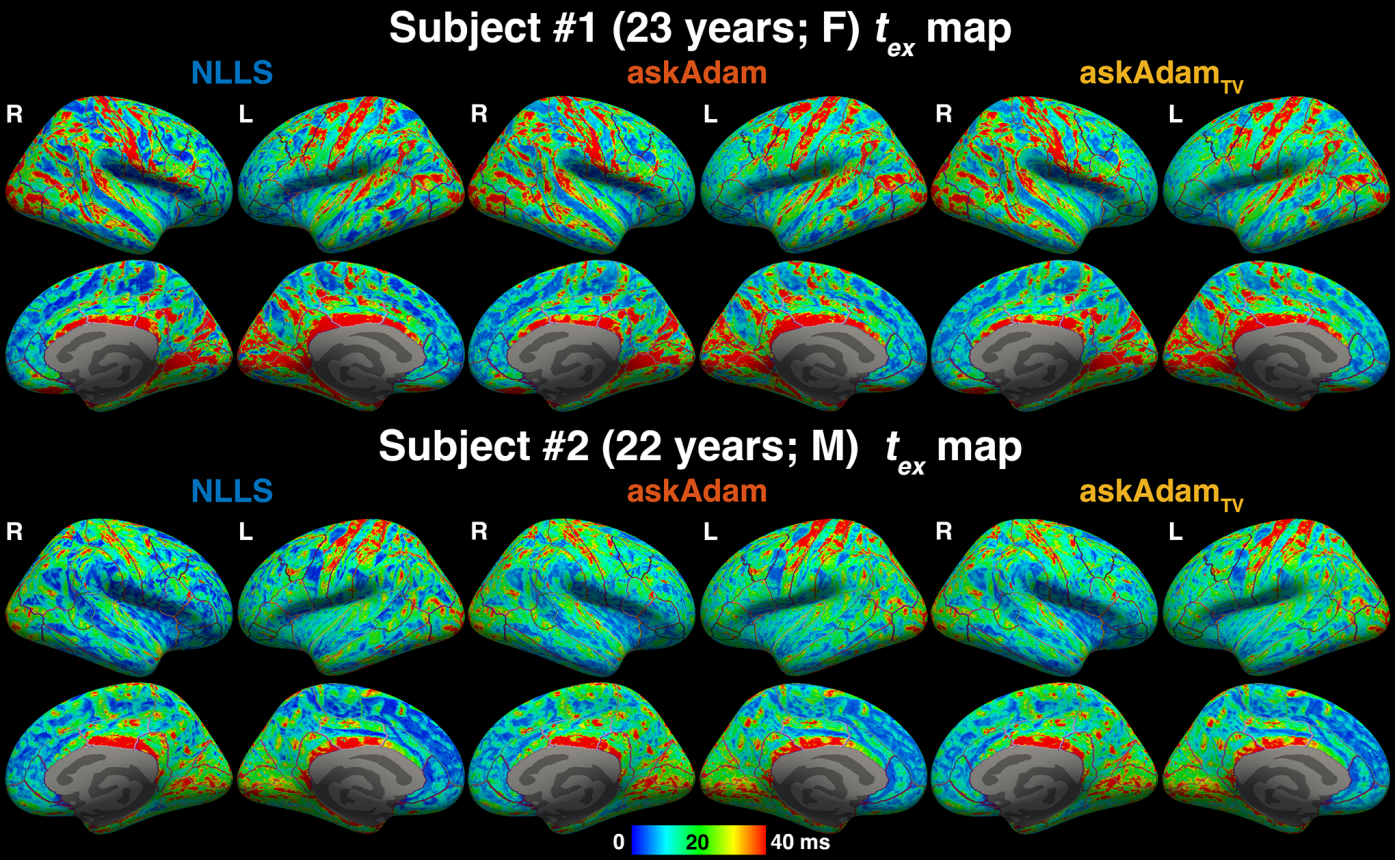
Examples of apparent exchange time$t_{ex}$maps projected at middle cortical depth, halfway between the pial surface and gray–white matter interface in two subjects (Subject #1: 23 years; female; Subject #2: 22 years; male). The outline on the cortical surface indicates the boundaries of the SynthSeg parcellation labels.

**Fig. 8.**
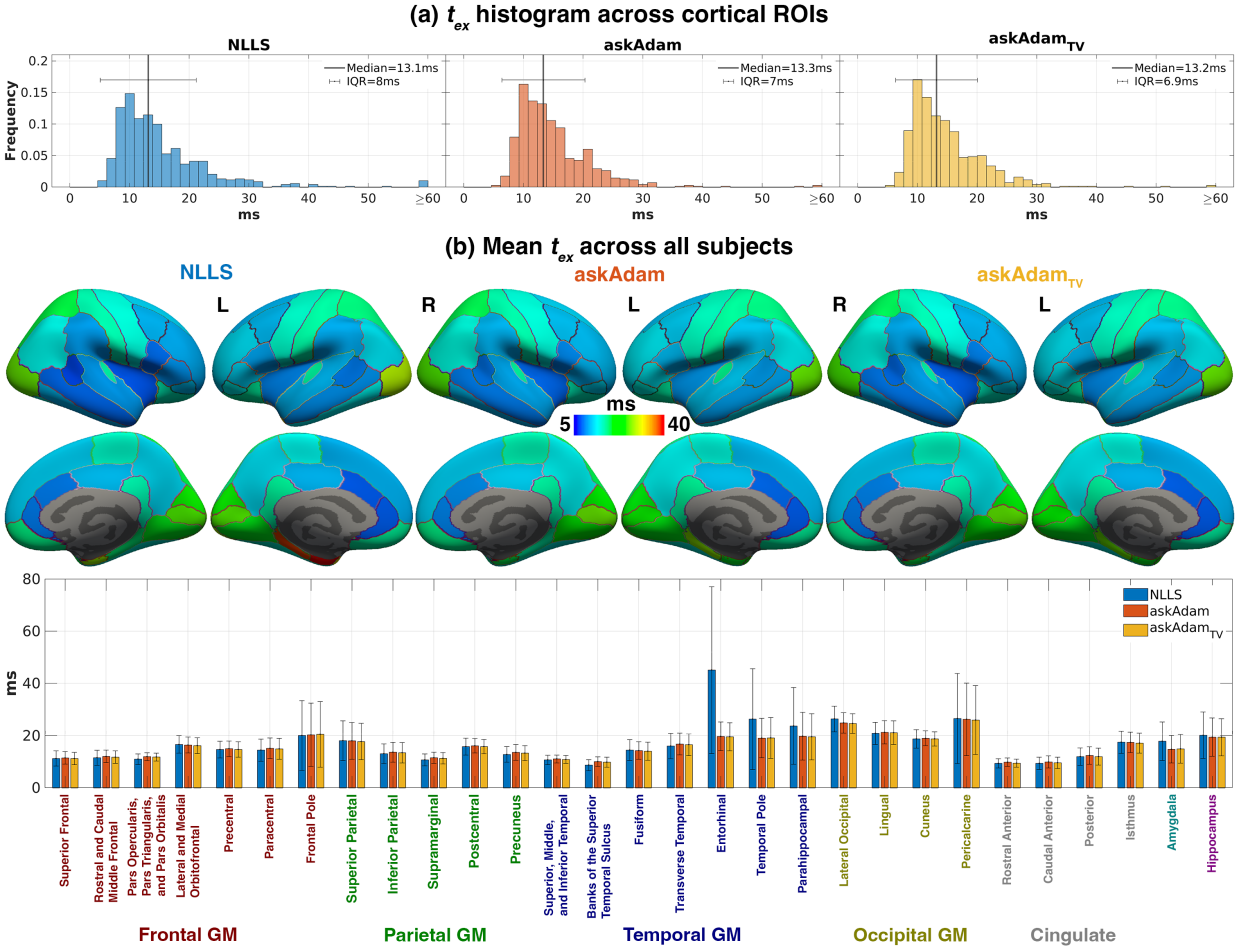
(a) Exchange time$t_{ex}$across all cortical ROIs and all subjects using the high SNR Connectome 2.0 dataset. Solid line indicates the median exchange time of the corresponding histogram. (b) Mean and standard deviation of the exchange time across all subjects for each cortical ROIs and their corresponding projection on the cortical surface.

In the group-level analysis, the median ± IQR exchange times estimated using NLLS, askAdam, and askAdam_TV_were 13.1 ± 8 ms, 13.3 ± 7 ms, and 13.2 ± 6.9 ms across all cortical ROIs for all subjects on the high SNR Connectome 2.0 data (Fig. 8a). Like the spatial patterns observed at the individual level, the frontal pole, pre-/post-central gyri, visual cortex, and posterior cingulate cortex had relatively longer exchange times than other ROIs on Connectome 2.0 (Fig. 8b). A spatial gradient of longer apparent exchange times was found moving from the anterior to the posterior cingulate cortex (Fig. 8b, c). The mean exchange times and standard deviations across subjects were similar for all three solvers, except for the fontal pole, entorhinal, temporal pole, and parahippocampal gyrus, where the NLLS solver estimated generally longer apparent exchange times and showed larger standard deviations (Fig. 8c).

**Fig. 9.**
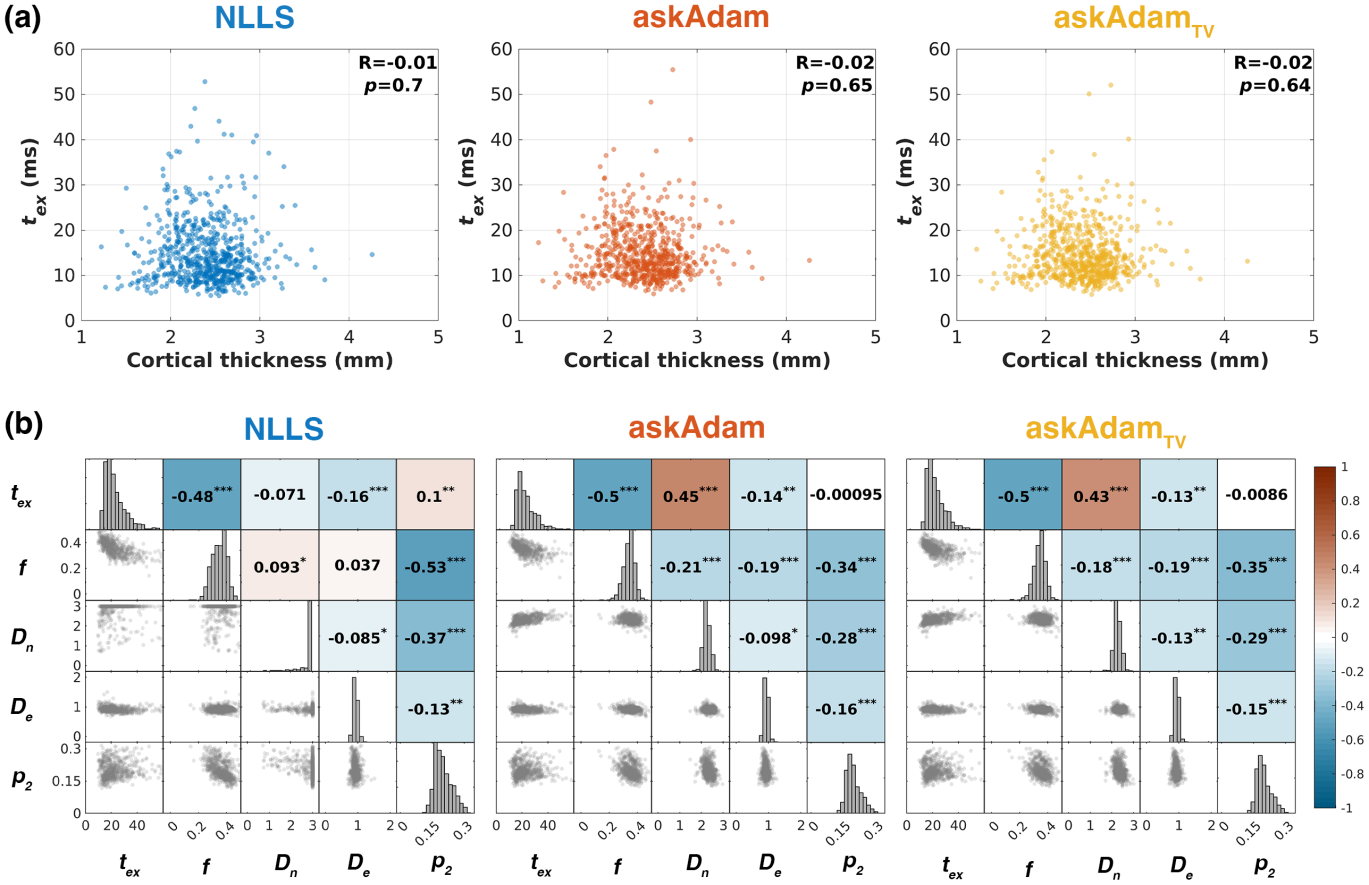
(a) Scatter plots of exchange time$t_{ex}$versus cortical thickness across all cortical ROIs and all subjects, with Pearson’s correlation coefficient R and*p*-value are also reported. (b) Correlation matrix between the NEXI microstructure parameters for each solver. *p-value < 0.05; **p-value < 0.01; ***p-value < 0.001.

To investigate potential partial volume effects from CSF and superficial white matter on NEXI model fitting, we performed a Pearson’s correlation analysis between the estimated exchange time and cortical thickness for all cortical ROIs and all subjects. No significant correlations were found between these two parameters on all solvers (R = -0.01; R = -0.02, and R = -0.02 for NLLS, askAdam, and askAdam_TV_,Fig. 9a). Among the NEXI tissue parameters, moderate correlations were found between$t_{ex}$andf(-0.48, -0.50, -0.50), between$t_{ex}$and$D_{n}$(-0.45, -0.43) for the askAdam solvers, and between$p_{2}$andf(-0.53, -0.34, -0.35) for NLLS, askAdam, and askAdam_TV_. Additionally, moderate correlations were observed between$p_{2}$and$D_{n}$(-0.37) for NLLS (Fig. 9b), whereas weaker correlations between these two parameters (-0.28, -0.29) were observed for askAdam and askAdam_TV_(Fig. 9b).

## 5 Discussion

In this work, we studied the apparent intercompartmental exchange times between neurites and extracellular water in the living human brain using the NEXI model fitted to data acquired on the state-of-the-art Connectome 2.0 scanner, which enabled us to obtain high-quality, whole-brain dMRI data at strong diffusion weighting (*b*-values up to 17.5 ms/μm^2^) and short diffusion time (*t_min_*= 13 ms). Similar to previous animal studies (Jelescu et al., 2022;Olesen et al., 2022), we observed that the zeroth-order rationally invariant (spherical mean) dMRI signals decreased with diffusion times at each*b*-value across the cortex, which was considered as the signature of intercompartmental exchange in gray matter (Olesen et al., 2022). While the measured exchange time across the human cortex using the Connectome 1.0 protocol agreed with the literature with a similar setup (Uhl et al., 2024), the apparent exchange time derived from the Connectome 2.0 protocol was substantially shorter, with median ± IQR of approximately 13 ± 8 ms. The apparent exchange time was observed to vary across the cortical ribbon. Furthermore, we introduced a novel processing tool to accelerate NEXI model fitting by more than 100-fold utilizing the latest GPU computing technology with comparable accuracy and improved precision compared with conventional voxel-wise NLLS fitting.

### 5.1 Comparison between Connectome 1.0 and Connectome 2.0 protocols

We evaluated the apparent inter-compartmental exchange time in the living human brain at the individual level using the ultra-high-gradient performance Connectome 2.0 scanner. The gradient system of the Connectome 2.0 scanner is five times more powerful than its predecessor Connectome 1.0 (considering together a magnetic field gradient 1.67 times stronger and 3 times faster than Connectome 1.0). This allows the Connectome 2.0 scanner to achieve the shortest diffusion time of 13 ms instead of 21 ms as Connectome 1.0 with similar diffusion weighting, facilitating access to experimental conditions better suited to probing fast water exchange when the two water compartments are less well mixed. Under the ideal conditions (i.e., with Gaussian noise or the noise statistics considered in the signal model), our simulations suggested that both Connectome 1.0 and Connectome 2.0 protocols can perform equally well (Fig. 2c). The advantages of the shorter diffusion times accessible on Connectome 2.0 are evident when the exchange time is relatively short (≤20 ms). Compared with the Connectome 1.0 protocol, the estimation of exchange time ≤20 ms using the Connectome 2.0 protocol was more precise and accurate, especially when the noise floor was not accounted for in the signal model (left panel,Fig. 2c). This observation is supported by the*in vivo*exchange time comparison between the two protocols (Fig. 5), where the Connectome 1.0-derived apparent exchange time is 2.67 times longer than that of the Connectome 2.0 using the ordinary NEXI model, driven by the reduced estimations in bothfand$r_{n}$, whereas the Connectome 1.0 estimations using the NEXI with Rician mean model became substantially shorter and more comparable with the Connectome 2.0 protocol results, potentially due to the incomplete removal of the noise floor by the denoising method. Note that our Connectome 1.0*in vivo*apparent exchange times derived from the ordinary NEXI model were comparable with the results reported in another study using the same method (103.9 ms using the ordinary NEXI model and 42.3 ms using the NEXI with Rician mean model) (Uhl et al., 2024). Besides the noise floor effect, another hypothesis that may account for the observed differences in apparent exchange times measured on Connectome 2.0 versus Connectome 1.0 could be that water exchange occurs across a broad range of times, as suggested in a recent report (Cai et al., 2023). As a result, using shorter diffusion times may sensitize the measurement of intercompartmental water exchange to the fast exchange process. Previously, shorter exchange times of 3–10 ms were observed in the*ex vivo*rat brain using the SMEX model in an experiment that used relatively short diffusion times (7.5–16 ms) (Olesen et al., 2022). We acknowledge that it is challenging to compare our results directly with those in the*ex vivo*rat brain due to potential interspecies differences in membrane permeability, tissue constituents, and water transport mechanisms caused by experiment conditions such as fixation and tissue preparation (Williamson et al., 2023). Differences related to the dMRI acquisition protocol and hardware, including the diffusion times, diffusion weightings, and SNR, can also introduce additional variations observed between the two studies. However, Veraart et al. estimated exchange times of 10–30 ms in the*in vivo*human brain gray matter using the anisotropic Kärger model (Veraart et al., 2020), and Williamson et al. measured an exchange time of 10 ms in the living mouse spinal cord via 2-dimensional diffusion exchange spectroscopy (Williamson et al., 2019,^2023^), which are both in agreement with our Connectome 2.0 measurements. Other imaging factors, such as SNR, may also affect the measured values. As shown in the noise propagation analysis, the (residual) Rician noise floor can lead to an overestimation of the exchange time (Fig. 2c). Even for Gaussian noise, low SNR conditions can lead to slightly overestimated exchange times as indicated by the*in silico*head phantom simulations (Fig. 3). Therefore, extra care may be needed on data processing and result interpretation when performing NEXI on standard clinical scanners as the gradient pulse duration, minimum achievable diffusion time, and SNR will not be comparable with high-gradient performance systems such as Connectome 1.0 or Connectome 2.0.

### 5.2 Implications on noise propagation analysis

Our noise propagation results indicate that the narrow pulse approximation utilized in the NEXI model adequately captures the exchange time given the clinically relevant SNR with high gradient performance setups. This observation remains consistent across different gradient pulse durations (6–10 ms). Although the SMEX model provides a more precise representation of the exchange dMRI signals, its direct usage on NLLS fitting could be relatively challenging due to the longer computational time required for solving the associated ordinary differential equations, especially for whole brain data. Accelerating SMEX forward signal generation using techniques such as artificial neural networks may be valuable to further improve the estimation performance in exchange time measurement in the future. One limitation of our simulation is that we did not take the restricted diffusion from the soma compartment into account for the forward signal simulation, which is also affected by the change of diffusion encoding gradient duration. In contrast, the Rician noise floor correction has a more notable effect on the accuracy and precision of exchange time estimation. Incorporating the Rician noise model into the NEXI signal model or having Gaussian noise present in the data minimizes estimation bias, aligning with findings from prior research (Uhl et al., 2024). Estimating noise levels in the data is crucial for implementing the NEXI model of Rician mean, achievable through methods such as MP-PCA noise level estimation (Veraart et al., 2016) or through the temporal statistics on multiple*b*= 0 images (assuming the noise level is constant over time). Alternatively, techniques such as Rician-bias-corrected MP-PCA denoising (Tournier et al., 2023) or using real-value dMRI data (Eichner et al., 2015;Fan et al., 2020;Tian et al., 2022) can reduce the Rician noise floor and potentially achieve noise statistics closer to Gaussian noise conditions. However, it is of the utmost importance to ensure the noise floor is eliminated by using these approaches, as the estimated apparent exchange time is highly sensitive to the residual noise floor, as illustrated inFigures 2cand5. In our extended analysis, results suggest that higher-order rotational invariants of dMRI signals may not contribute significantly to NEXI microstructure parameter estimation (Fig. 3), likely due to the highly disperse nature of neurite orientations in gray matter. In most cases, the zeroth-order signals (spherical mean) are sufficient for estimating the apparent exchange time. Nevertheless, incorporating the second-order signals introduces an additional microstructure parameter ($p_{2}$), representing neurite orientation dispersion, without sacrificing estimation performance (see alsoSupplementary Fig. S4andTable S1for*in vivo*comparison), which could be relevant in disease contexts.

### 5.3 Effects of diffusion weighting on estimated exchange time

We utilized strong diffusion weighting (maximum*b*-values of 17.5 ms/μm^2^) for NEXI to ensure water exchange can be sampled when the hindered diffusion of extracellular water does not contribute to the diffusion signal. To gain further insight into whether such strong diffusion weighting is needed to measure the exchange time accurately, we performed a retrospective analysis by repeating the ROI-averaged NEXI estimation using dMRI data limited to*b*= 6.5 ms/μm^2^. While the apparent exchange times remain comparable in frontal, parietal, and occipital gray matter regions, reductions in apparent exchange time are evident in the temporal gray matter, amygdala, and hippocampus when high*b*-value data are excluded (refer toSupplementary Fig. S7andTable S2). This decrease in$t_{ex}\;=(1-f)/r_{n}$is attributed to the apparent overestimation of the intra-neurite volume fractionf(in contrast to using the full*b*-value range), with the estimated apparent exchange rates$r_{n}$from neurites to the extracellular space remaining consistent across both datasets. Note that using the Standard Model without considering water exchange in grey matter may lead to underestimation of the neurite volume fraction, especially with long diffusion times (seeSupplementary Fig. S9).

### 5.4 *In vivo* apparent exchange time across cortical ribbon

Interestingly, the measured apparent exchange times exhibit spatial variability across the cortex, showing a pattern akin to cortical myelination (Edwards et al., 2018;Marques et al., 2017) derived from multimodality images, as well as to the previous*in vivo*NEXI study (Uhl et al., 2024). Specifically, regions with higher levels of myelination, such as the motor cortex, somatosensory cortex, visual cortex, transverse temporal gyrus, and posterior part of the cingulate cortex, tend to display longer apparent exchange times. Additionally, there appears to be a spatial gradient of myelination increasing from the anterior to the posterior parts of the cingulate cortex, consistent with the observed pattern in the apparent exchange time projection illustrated inFigure 8. Further investigation is needed to elucidate the association between cortical myelination and apparent intercompartmental exchange times.

### 5.5 Accelerating NEXI processing

We have also demonstrated a significant acceleration of NEXI model fitting, achieving over 100-fold increase in speed using the GPU-based askAdam framework while maintaining comparable estimation performance. Our evaluation using the*in silico*head phantom revealed that, although askAdam’s estimation accuracy is slightly reduced compared with voxel-wise NLLS fitting, the bias in apparent exchange time introduced by askAdam is small (difference on median = 0.41 ms at SNR = 50 between NLLS and askAdam,Fig. 4), particularly when considering inter-subject variability (Fig. 8). Another advantage of askAdam is its ability to integrate spatial regularization by updating all microstructure parameters simultaneously during the fitting process. This feature proves particularly beneficial when dealing with low SNR data. As a proof-of-concept demonstration, we implemented 2D-TV regularization on the neurite volume fraction parameter map (seeSupplementary Fig. S2). Yet, more advanced methods, such as total generalized variation (TGV) (Bredies et al., 2010;Knoll et al., 2011) or structure-guided TV (Ehrhardt & Betcke, 2016), can also be employed. These methods have demonstrated better preservation of anatomical structures, which could be important for cortical gray matter given its highly folded nature. Moreover, the flexibility of askAdam’s loss function allows spatial regularization to extend beyond a single parameter map. It can be applied simultaneously across multi-parameter maps along with other anatomical information, such as sharp edges in the image (Liu et al., 2011). Despite spatial regularization may be useful in reducing the spatial variance and stabilizing the estimation, caution should be exercised to determine the optimal regularization parameter to prevent measurement bias resulting from over-regularization (over-smoothing) that could potentially decrease the sensitivity of the method in assessing specific pathologies, as diseased tissues often have greater microstructural variations than normal tissues. Nonetheless, spatial regularization is only an optional feature of askAdam and can be disabled in the optimization. As shown inFigure 8, the estimation performance without TV regularization is nearly identical to the results obtained with TV regularization at the group-level comparison. It is worth noting that the primary benefit of utilizing the “askAdam” framework lies in its computation time efficiency. Since the estimation principle is still based on minimizing the signal differences between a tissue model and the measurement data, it will inherit similar degeneracy issues in ill-conditioned parameter estimation as in NLLS voxel-wise approaches.

### 5.6 Limitations

Our study has some limitations. First, the NEXI model employed in our analysis does not explicitly account for water diffusion within the soma. It is established that the soma contributes approximately 10–20% of the water signal at low*b*-values in gray matter (Olesen et al., 2022). At weak diffusion weighting, the signal contribution from the soma may be non-negligible. We observed markedly shorter measured apparent exchange times when incorporating*b*= 1 ms/μm^2^in the NEXI fitting (Supplementary Table S2). The fit residuals also exhibited stronger systematic differences between the NEXI forward signal and the measurements (seeSupplementary Fig. S6), prompting us to include data with only*b*> 1 ms/μm^2^for the NEXI fitting. Note that the*in vivo*data demonstrated only weak time dependence at*b*= 1 ms/μm^2^(seeSupplementary Figs. S5 and S6). This suggests that at the low diffusion weightings, it will be more challenging to disentangle the exchange effect from diffusion. Expanding the NEXI model to encompass a non-exchanging restricted compartment to represent the soma could potentially alleviate signal discrepancies at low*b*-values (Olesen et al., 2022), yet this would introduce additional model parameters that may compromise estimation precision. In contrast, signal originating from myelinated fibers in gray matter that is not accounted for in the NEXI model, though comprising only a small portion (Shapson-Coe et al., 2024), may become more pronounced at very high*b*-values (e.g., ≥17 ms/μm^2^for*in vivo*) and introduce biases in the NEXI estimation (Olesen et al., 2022). An*ex vivo*mouse study at 16.4T byOlesen et al. (2022)demonstrated that the differences in exchange time with and without considering the myelinated fiber were between 0.3 and 0.9 ms with the measured exchange times between 3.9 and 5.8 ms. Whether these differences hold for*in vivo*imaging will need further investigation. However, it will be particularly challenging to separate the myelinated fiber signal from the unmyelinated neurite signal in gray matter*in vivo*human experiments, considering the SNR and*b*-values demands. Other signal sources, including microscopic kurtosis (Henriques et al., 2021), effects of the localization regime (Moutal & Grebenkov, 2020), effects of deviation from idealized geometries (Ianus et al., 2021), and effects of dendritic spines (Palombo et al., 2017) among others, that were not accounted for in the NEXI signal model may also contribute to the apparent exchange time biases we observed between the Connectome 1.0 and Connectome 2.0 protocols. While intra-vascular and extra-vascular signals were considered to be highly suppressed due to the use of high*b*-value data here, the exchange effect between these two compartments may also need to be considered when low*b*-value (<1 ms/µm^2^) data are used to fit the model with soma compartment.

Another limitation of NEXI is that the signal model does not account for the potential transverse relaxation time differences between the two compartments. Several reports have suggested that the intra-neurite transverse relaxation time is slightly longer than that of the extra-cellular water (Gong et al., 2024;Lee et al., 2024;Veraart et al., 2018). This difference can lead to an echo time dependence on the estimated apparent neurite volume fraction, thus introducing an additional study bias in the estimated apparent exchange time when different echo times are used in the acquisition. This effect may be alleviated by incorporating the compartmental transverse relaxation rates in the NEXI model with dMRI data acquired at multiple echo times (Lee et al., 2024). The additional compartmental relaxation parameters may also provide extra insight into the microstructure environment, but this will certainly lengthen the scan time of the existing protocol due to additional data acquisition.

The drawback of including only higher*b*-value (>1 ms/μm^2^) data is the reduced sensitivity to estimate the neurite diffusivity. Previous studies demonstrated that the neurite diffusivity was about 2 μm^2^/ms in white matter (Coelho et al., 2022;Dhital et al., 2019) and gray matter (Ianuş et al., 2022;Palombo et al., 2020), which required lower*b*-value data for a reliable estimation. This is in line with our simulation results and*in vivo*data where the neurite diffusivity is the least robust estimated parameter in the NEXI model given the current imaging protocols and often fitted to the upper bound of the allowed range. Setting a higher fitting upper bound for neurite diffusivity only shows minor impacts on the estimated apparent exchange time (seeSupplementary Fig. S3). Further investigation is necessary to accurately assess soma properties in conjunction with the intercompartmental exchange effect.

The partial volume effect arising from the superficial white matter and CSF due to the relatively large voxel size compared with cortical thickness can also affect the measurement accuracy (our dMRI data: 2 mm isotropic vs. typical cortical thickness = 2.2–2.9 mm across all ages;Frangou et al., 2022). We examined the potential impact of the partial volume effect in our data through correlation analysis between the exchange time and cortical thickness and found no significant association between the two parameters (Fig. 9a). While this evidence may suggest that the partial volume effect due to local white matter and CSF may not influence the measured apparent exchange time significantly, it is also possible that they could have an opposite effect in the NEXI estimation. Further investigation is required to understand how different tissue types (gray matter, white matter, and CSF) coexisting in a voxel may impact the estimation of the*in vivo*NEXI exchange time. Increasing the spatial resolution can mitigate the partial volume effect at the expense of reducing SNR and lengthening scan time. Nevertheless, given the high-quality data provided by the Connectome 2.0 scanner (SNR = 55 at*b*= 0 before denoising), obtaining high-resolution dMRI while maintaining reasonable SNR using standard spin-echo EPI sequences may be feasible. Alternatively, recent advancements in pulse sequence development (Dong et al., 2024;Ramos‐Llordén et al., 2020;Setsompop et al., 2018), denoising (Henriques et al., 2023;Lemberskiy et al., 2023;Olesen et al., 2023), super-resolution techniques (Coupé et al., 2013;H. H. Lee et al., 2019;Manjón et al., 2010), and diffusion sampling strategy (Fan et al., 2017) for high-resolution diffusion imaging offer a promising avenue to achieve high resolution with good SNR for gray matter imaging. Another advantage of higher spatial resolution is the potential to investigate the intercompartment exchange effect at multiple cortical depths, providing more specific information reflecting tissue composition and arrangement across the cortical layers.

To be able to obtain high-quality data for NEXI exchange time mapping, we employed a protocol comprising 15*b*-values at 64 diffusion gradient directions from 3 diffusion times, resulting in more than 80 min total acquisition time, which is clearly too long for clinical applications. Reducing the number of diffusion gradient directions by half as the protocol used for Connectome 1.0 and Connectome 2.0 comparison can shorten the scan time to 40 min while retaining comparable results as the 64-direction high SNR protocol. Future studies will focus on protocol optimization on sequence parameters, such as diffusion times,*b*-values, and number of diffusion gradient directions, to achieve a more accessible scan time (<20 min) for clinical and research applications.

## 6 Conclusions

The ultra-high-gradient performance Connectome 2.0 scanner provides high-quality data and greater flexibility in experimental design to measure intercompartmental exchange effects between intracellular and extracellular water in the brain gray matter. The*in vivo*apparent intercompartmental exchange time was estimated to be about 13 ± 8 ms across the human cortex based on the anisotropic Kärger model. Our work further indicated that addressing the Rician noise floor sufficiently, either in the signal model or in the data processing, is crucial to measure the short exchange time (≤20 ms) using NEXI, especially when a short diffusion time cannot be easily deployed due to hardware constraints. The narrow pulse approximation in NEXI did not introduce significant bias in the apparent exchange time measurements using (ultra-)high gradient performance MRI systems, such as Connectome 1.0 and Connectome 2.0 scanners, under clinically relevant SNR circumstances. Using the GPU-based askAdam processing pipeline, we accelerated the NEXI data fitting process by 100-fold. Our work provides additional insights and groundwork that may aid in NEXI protocol optimization for*in vivo*imaging in future studies.

## Supplementary Material

Supplementary Material

## Data Availability

The data and code that support the findings of this study will be made available upon reasonable request. The GPU processing tool for the ordinary NEXI model and NEXI with Rician mean model parameter estimation is available athttps://github.com/kschan0214/gacelle.
