# Supplementary material for: In vivo human neurite exchange time imaging at 500 mT/m diffusion gradients
Source: Imaging Neurosci (Camb). 2025 Apr 22;3:imag_a_00544. doi: 10.1162/imag_a_00544 (PMC12302768; doi:10.1162/imag_a_00544)
Supplement: Supplementary Material [file imag_a_00544-supp.pdf]

Supplementary Materials

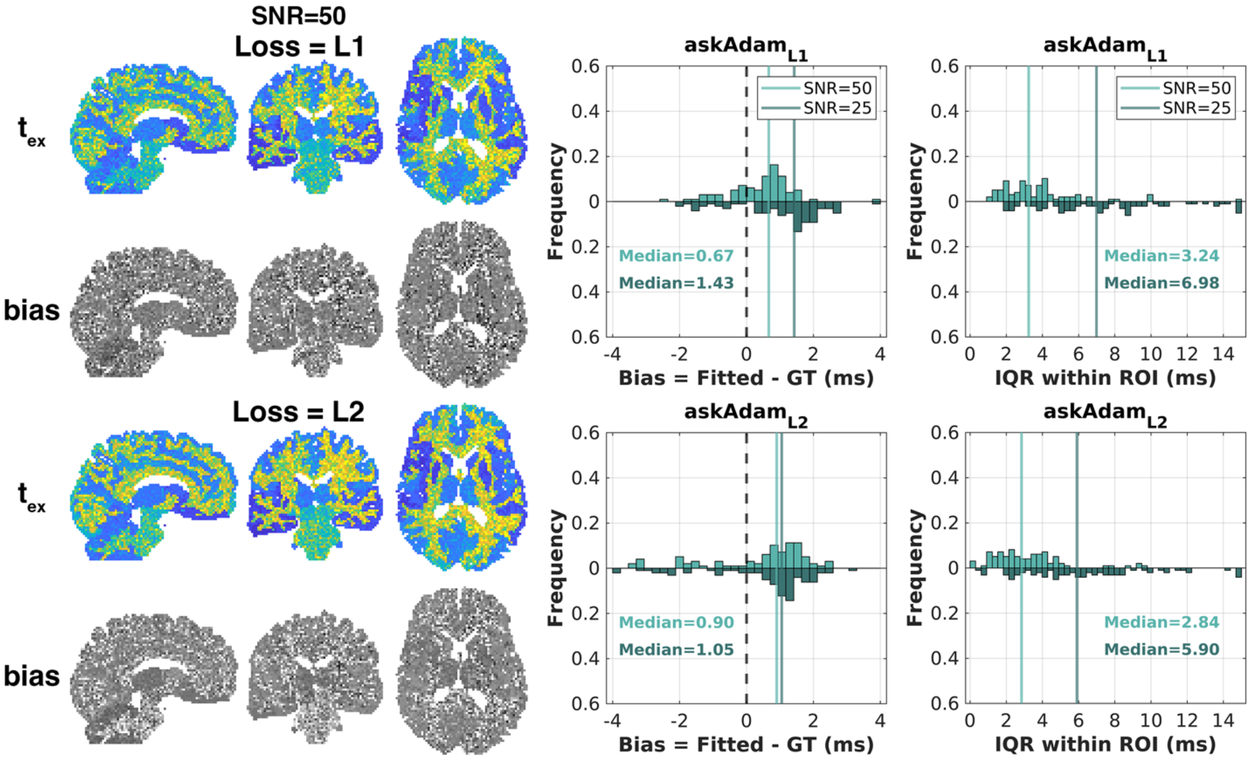

Figure S1: The same result shown in Figure 3 using askAdam solver with (top) L1-norm and (bottom) L2=norm as the loss function as provided in Eq. (7). The L1-norm loss function produces more accurate results at SNR=50, whereas the results of L2-norm are more accurate at SNR=25. The t_{ex} estimation precision is slightly better with the L2-norm. Given our in vivo data have SNR>50 at *b*=0, we decided to use the L1-norm as the loss function in this work.

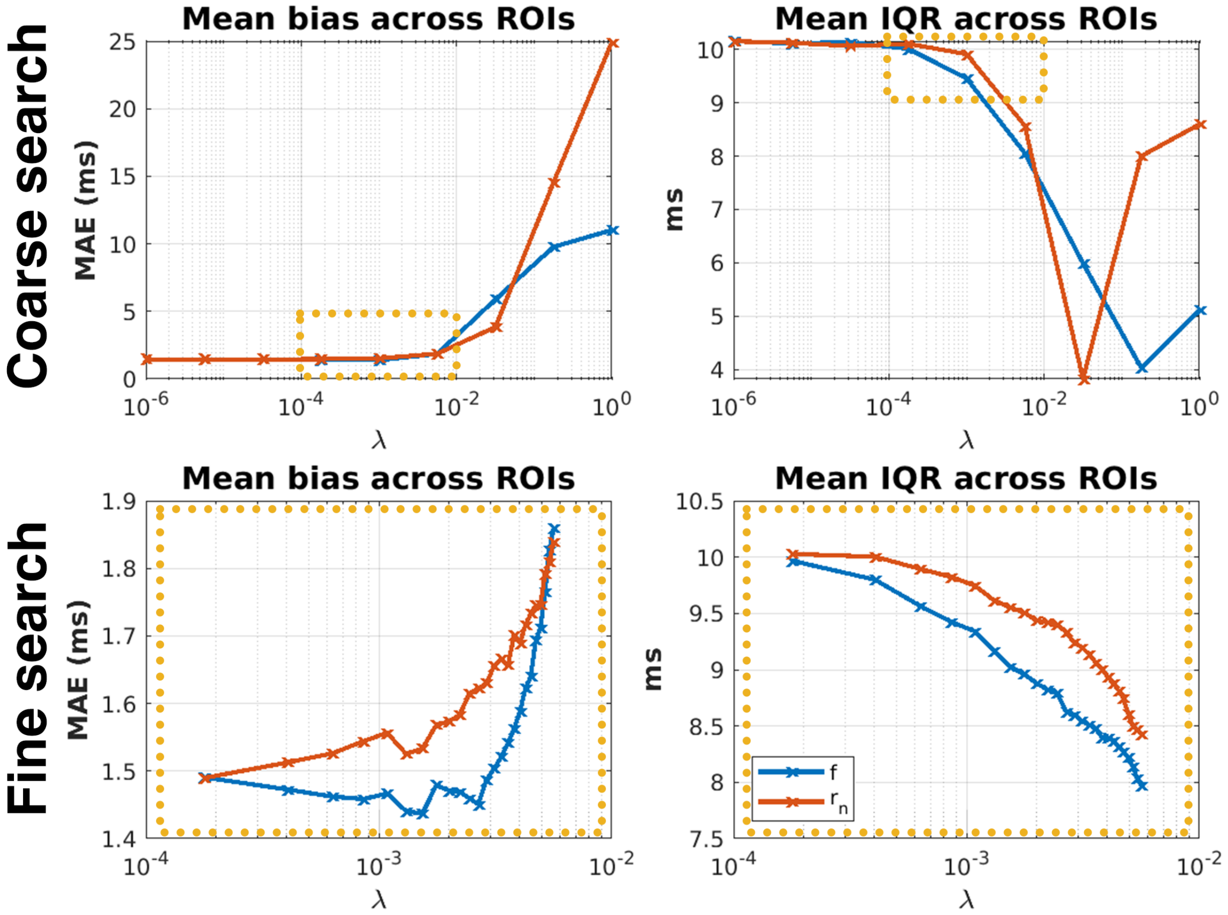

Figure S2: Determining the optimal regularization parameter and map for askAdam$_{TV}$ using *in silico* head phantom data at SNR=50. Regularization on two NEXI parameter maps (f and r_n) was studied over a range of regularization values. The optimal regularization values were determined in a two-step searching approach: firstly, a coarse search was performed in the range between 1e-6 and 1 with a non-linear step size to sample 9 different regularization values (top row). Mean absolute error (MAE) and the mean IQR of the exchange time across all ROIs were used to decide a narrower range (yellow box) for a finer search. The coarse search results suggested that the regularization is in a good balance of estimation accuracy and precision in the range between 1.8e-4 and 0.056 for both parameters. In the fine-search step, 25 regularization values were sampled over this range with a linear step size of 2.3e-4 (bottom row). Across this narrow range when the estimation accuracy has not deteriorated too severely, applying regularization on the f map is most always performed better than r_n (smaller bias and narrower IQR).

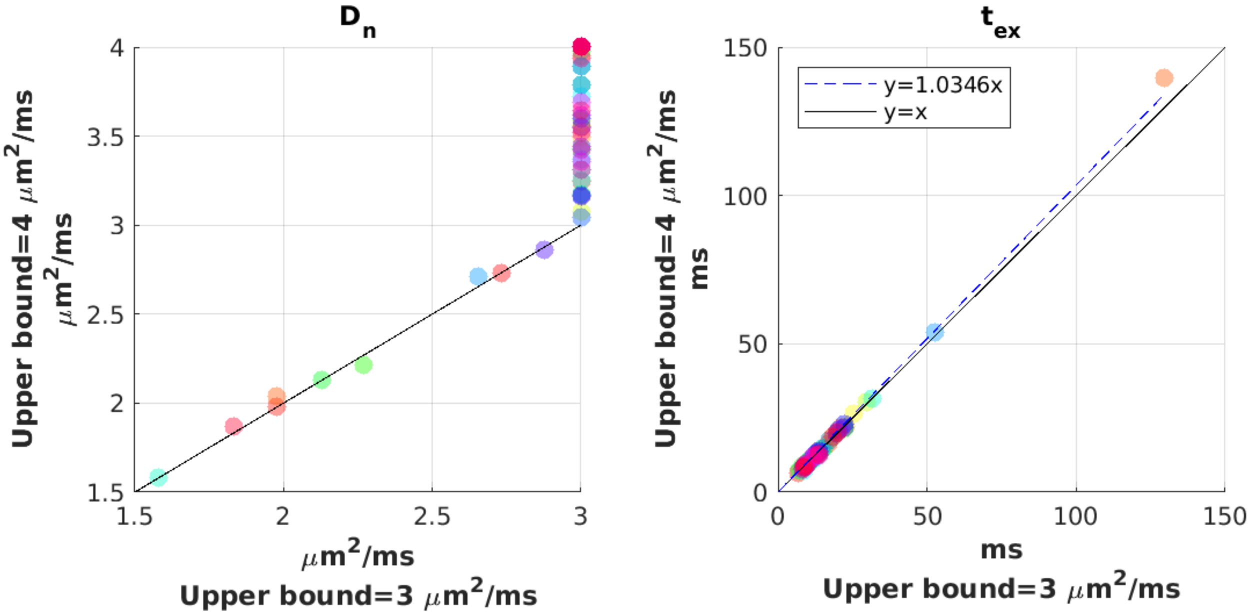

Figure S3: Scatterplots of (left) neurite diffusivity D_n and (right) exchange time t_{ex} by setting fitting upper bounds of neurite diffusivity at 3 and 4 μm^2/ms. Each data point represents the median value of an ROI.

We repeated the *in vivo* ROI fitting analysis described in Section 3.2.3 using zeroth and second-order rotationally invariant dMRI signals, respectively, on the Connectome 2.0 data to validate the aforementioned noise propagation analysis finding. Both $l_{max} = 0$ and $l_{max} = 2$ NEXI models showed similar fitting quality (dashed lines, Figure S3) on the zeroth order rotationally invariant $S_{l=0}$ signal. In this analysis, the exchange time t_{ex} ranged from 14.9 ms (frontal) to 33.5 ms (occipital) for the NEXI $l_{max} = 0$ model (Table S1). The values of the fitted parameters were generally similar between $l_{max} = 0$ and $l_{max} = 2$. In all ROIs, the intra-neurite diffusivity D_n was faster than the extra-cellular diffusivity D_e for either $l_{max} = 0$ or $l_{max} = 2$ model fitting.

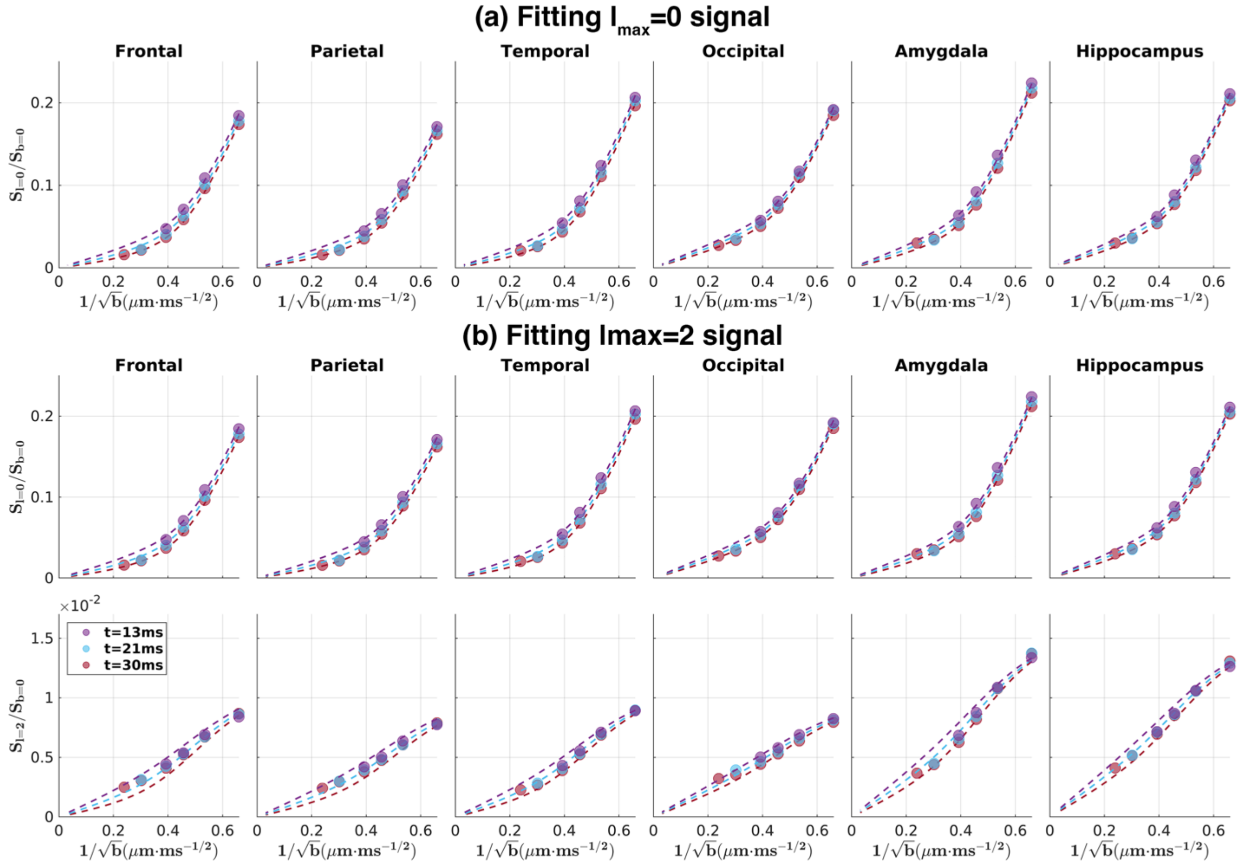

Figure S4: Results of NEXI model fitting on ROI-averaged *in vivo* dMRI signals using Connectome 2.0 protocol data. The NEXI model of $l_{max} = 0$ was fitted to (a) only the zeroth order rotationally invariant signals $S_{l=0}$, and the NEXI model of $l_{max} = 2$ was fitted to (b) both zeroth and second order rotationally invariant signals, $S_{l=0}$ and $S_{l=2}$. Circles represent the measured data and dashed lines represent the NEXI model fitting curve derived from the fitted tissue parameters.

Table S1: NEXI tissue parameters of grey matter ROI derived from ROI-averaged *in vivo* dMRI signals using Connectome 2.0 data.

	t_{ex} (ms)		f		D_n (μm^2/ms)		D_e (μm^2/ms)		p_2
	$l_{max} = 0$	$l_{max} = 2$	$l_{max} = 0$	$l_{max} = 2$	$l_{max} = 0$	$l_{max} = 2$	$l_{max} = 0$	$l_{max} = 2$	$l_{max} = 2$
Frontal	14.88	14.93	0.35	0.35	3.00	3.00	0.89	0.89	0.21
Parietal	17.79	17.84	0.32	0.32	3.00	3.00	0.94	0.94	0.21
Temporal	17.06	17.06	0.35	0.35	3.00	3.00	0.81	0.81	0.20
Occipital	33.45	33.51	0.34	0.34	3.00	3.00	0.89	0.89	0.19
Amygdala	23.00	22.96	0.37	0.37	3.00	3.00	0.77	0.77	0.28
Hippocampus	29.31	29.36	0.36	0.36	3.00	3.00	0.82	0.82	0.28

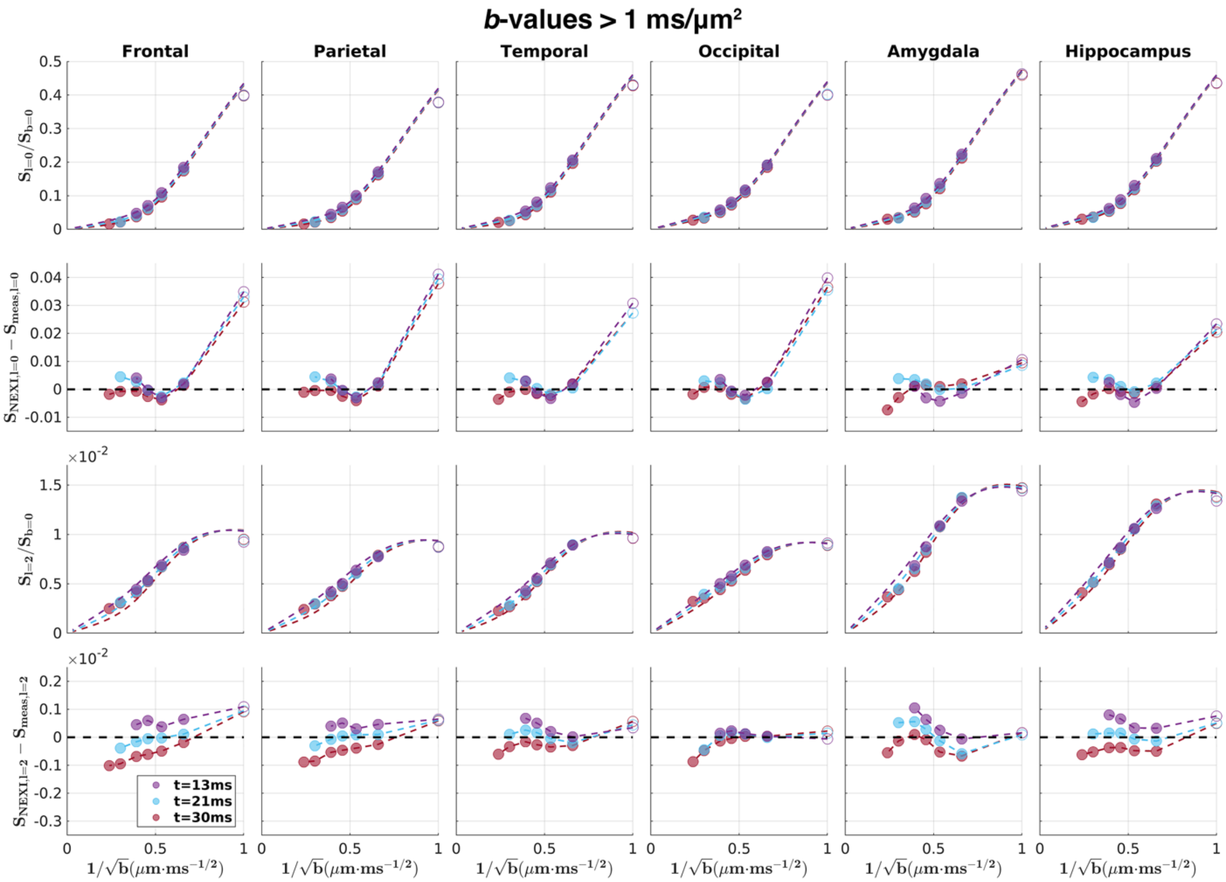

Figure S5: NEXI model fitting with ROI-averaged dMRI signals using $l_{max} = 2$ on Connectome 2.0 protocol data. This is the same result as shown in Figure 5a in the main text, but the forward simulated NEXI signal was extrapolated to b=1 ms/μm^2. Solid circles: measured data used in data fitting; hollow circles: measured data that was not used in data fitting. Dashed lines: forward NEXI signals derived from the fitted microstructure parameters.

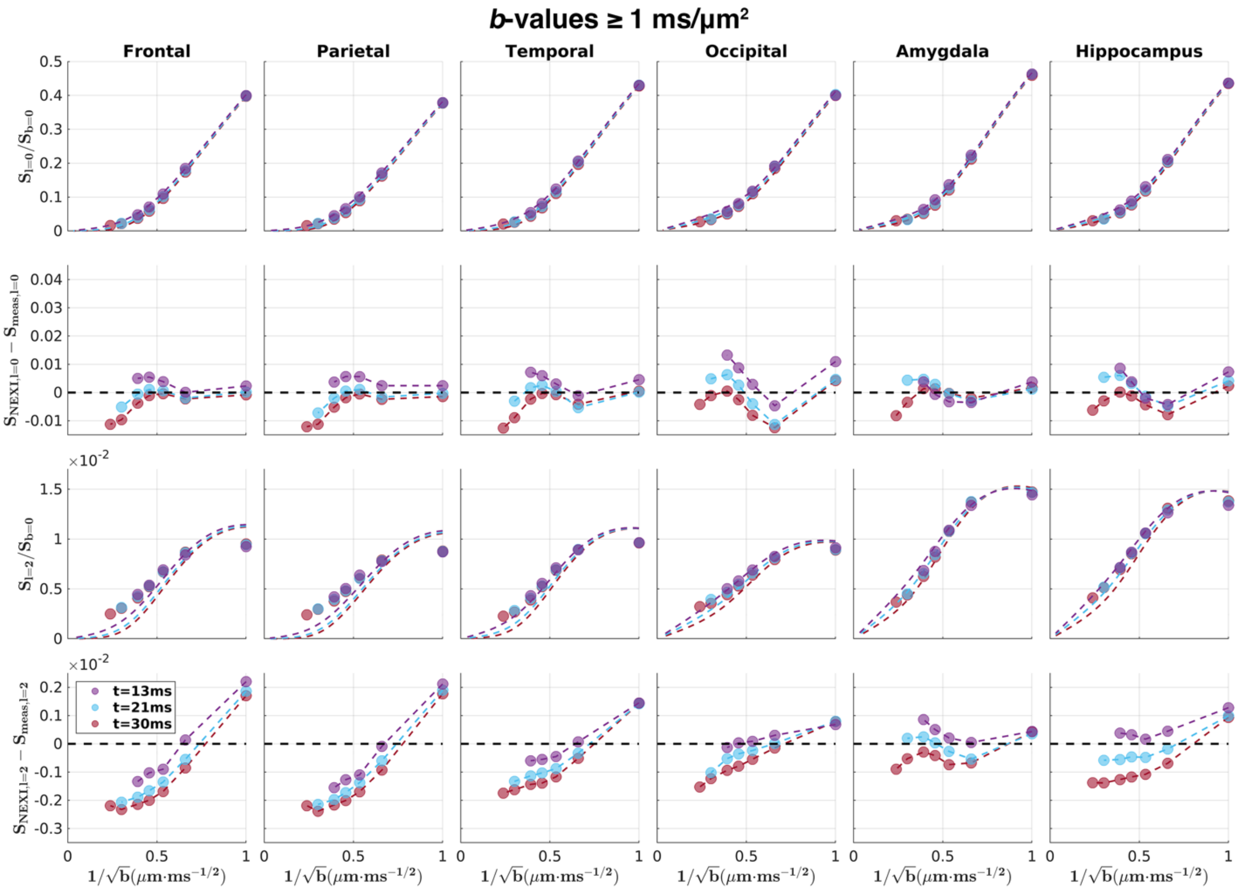

Figure S6: NEXI model fitting with ROI-averaged dMRI signals using $l_{max} = 2$ on Connectome 2.0 data. The results shown here were computed with the inclusion of data acquired at *b*=1 ms/μm^2. Solid circles: measured data used in data fitting. Dashed lines: forward NEXI signals derived from the fitted microstructure parameters.

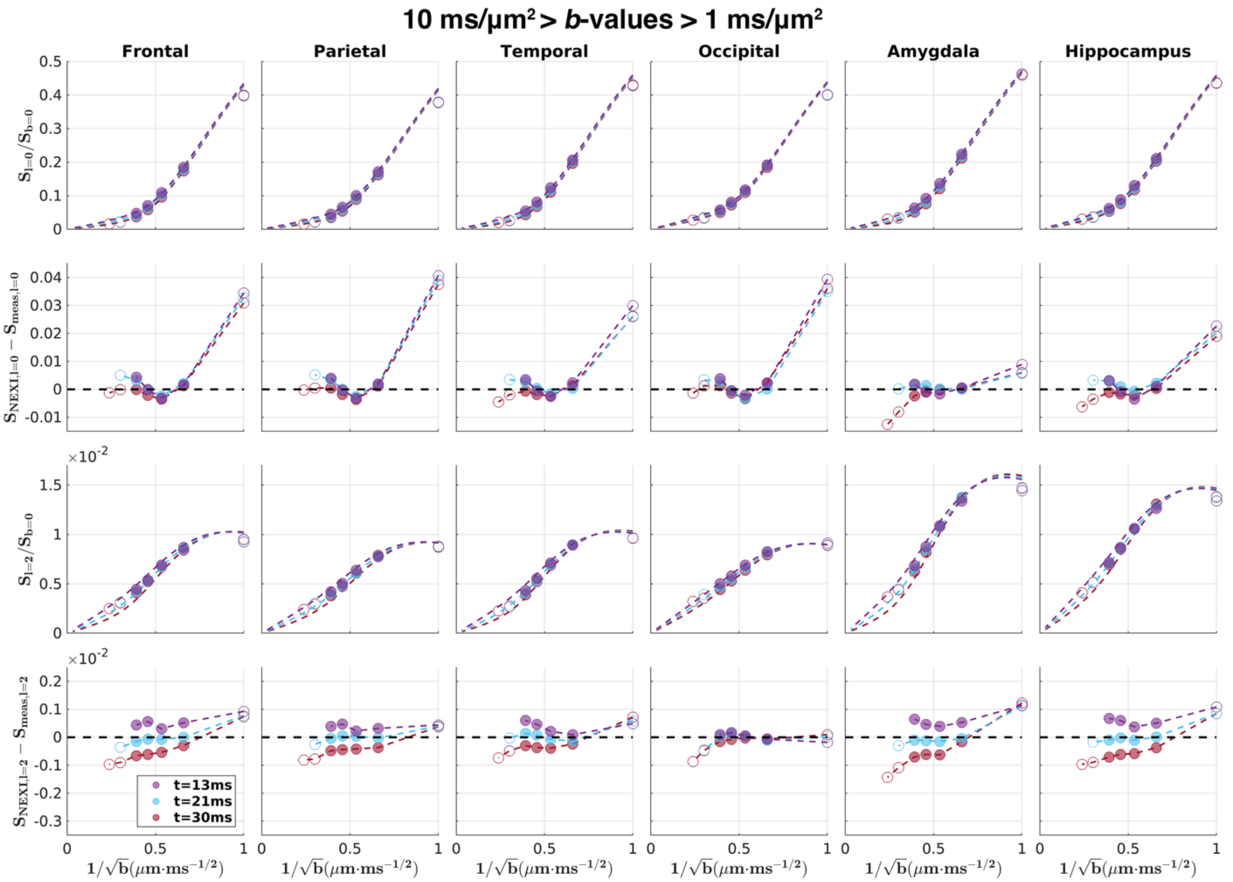

Figure S7: NEXI model fitting with ROI-averaged dMRI signals using $l_{max} = 2$ on Connectome 2.0 protocol data. The results shown here were computed when high b-value data were excluded in data fitting (b_{max}=6.5 ms/μm^2). Solid circles: measured data used in data fitting; hollow circles: measured data that was not used in data fitting. Dashed lines: forward NEXI signals derived from the fitted microstructure parameters.

Table S2: NEXI microstructure parameters of various grey matter ROIs derived from ROI-averaged dMRI signal using Connectome 2.0 protocol data with different *b*-values.

	t_{ex} (ms)		f		D_n (μm^2/ms)		D_e (μm^2/ms)		p_2	
	$b \geq 1$	$10 > b > 1$	$b \geq 1$	$10 > b > 1$	$b \geq 1$	$10 > b > 1$	$b \geq 1$	$10 > b > 1$	$b \geq 1$	$10 > b > 1$
Frontal	1.85	15.66	0.70	0.34	3.00	3.00	1.34	0.90	0.13	0.21
Parietal	1.13	19.35	0.78	0.31	3.00	3.00	1.89	0.94	0.12	0.21
Temporal	3.44	14.95	0.61	0.37	3.00	3.00	0.98	0.81	0.13	0.19
Occipital	14.30	34.66	0.48	0.34	3.00	3.00	1.07	0.89	0.15	0.19
Amygdala	17.76	12.90	0.40	0.42	3.00	3.00	0.79	0.77	0.26	0.26
Hippocampus	16.49	23.00	0.45	0.38	3.00	3.00	0.90	0.82	0.24	0.27

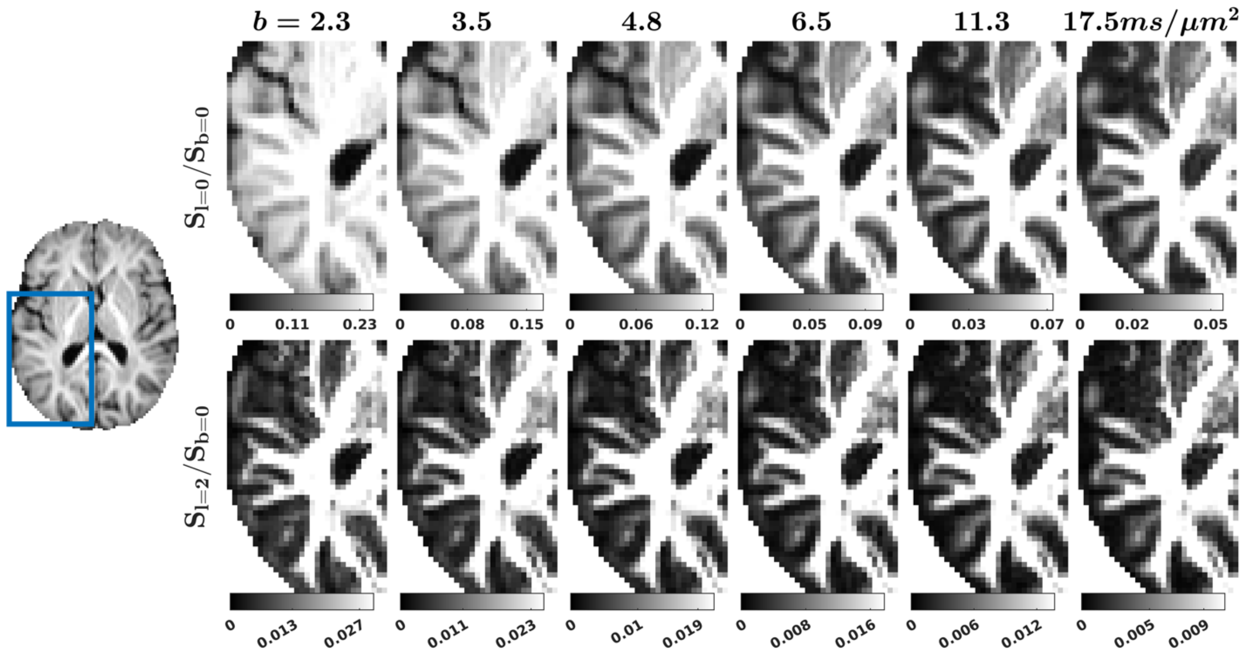

Figure S8: Zoom-in views of the zeroth and second order rotationally invariant dMRI signals at t of 30 ms and *b*-values from 2.3 ms/μm^2 to 17.5 ms/μm^2 on one subject from the high SNR Connectome 2.0 cohort on the same slice shown in Figure 6.

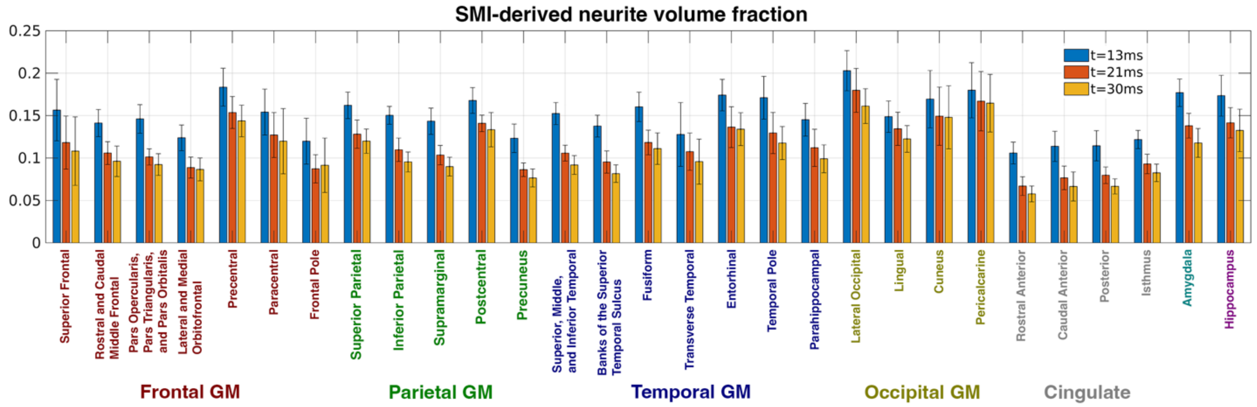

Figure S9: Neurite volume fraction across various cortical ROIs and across all subjects without considering the exchange effect on the Connectome 2.0 dataset. Voxel-wise estimation was performed using the Standard Model Imaging (SMI) toolbox (Coelho et al., 2022) on dMRI data acquired at three diffusion times, respectively.

Reference

Coelho, S., Baete, S.H., Lemberskiy, G., Ades-Aron, B., Barrol, G., Veraart, J., Novikov, D.S., Fieremans, E., 2022. Reproducibility of the Standard Model of diffusion in white matter on clinical MRI systems. NeuroImage 257, 119290. https://doi.org/10.1016/j.neuroimage.2022.119290
